# The artificial intelligence cooperative: READ-COOP, Transkribus, and the benefits of shared community infrastructure for automated text recognition

**DOI:** 10.12688/openreseurope.18747.2

**Published:** 2025-10-07

**Authors:** Melissa Terras, Bettina Anzinger, Paul Gooding, Günter Mühlberger, Michaela Prien, Joe Nockels, C. Annemieke Romein, Andy Stauder, Florian Stauder

**Affiliations:** 1 Edinburgh College of Art, The University of Edinburgh, Edinburgh, Scotland, EH8 9BT, UK; 2 READ-COOP, Innsbruck, Tirol, 6020 Innsbruck, Austria; 3 Information Studies, University of Glasgow, Glasgow, Scotland, G12 8QH, UK; 4 Digitalisierung und elektronische Archivierung (DEA), Leopold Franzens Universität für Innsbruck, Innsbruck, Tirol, 6020 Innsbruck, Austria; 5 Digital Humanities Institute, University of Sheffield, Sheffield, South Yorkshire, S3 7QY, UK; 6 Section Knowledge, Transformation & Society, KiTeS University of Twente, Faculty Behavioural, Management and Social Sciences (BMS), PO Box 217, 7500 AE Enschede, 1012 DK Amsterdam, The Netherlands

**Keywords:** Artificial Intelligence, Machine Learning, Handwritten Text Recognition, Automated Text Recognition, Digital Cultural Heritage, Innovation, Business Models, Cooperative Societies

## Abstract

**Background:**

Stakeholder input into Artificial Intelligence (AI) and Machine Learning (ML) is critical for creating AI systems that are both innovative and accountable. This paper examines READ-COOP ( https://readcoop.eu), a platform cooperative that develops and hosts its own AI and ML Automated Text Recognition (ATR) tools ( https://transkribus.org). This case study demonstrates an alternative cooperative governance model for technology innovation and creating responsible AI infrastructure.

**Methods:**

We employ Reflection-In-Action and a qualitative Member questionnaire to document the development of READ-COOP from European Commission funded research project to independent cooperative. We assess the cooperative’s structure, management, and community engagement from 2019 to 2024, including membership dynamics, use, governance, and operational efficacy.

**Results:**

As of October 2024, READ-COOP has 227 Members from 30 countries, and 235,000 registered User accounts. Transkribus has processed 90 million digital images of historical texts, demonstrating effective AI utilization in the cultural heritage sector, winning the European Union’s Horizon Impact Award 2020. The cooperative approach facilitates democratic decision-making, leading to sustainable growth, and significant stakeholder involvement. Qualitative feedback indicates high levels of satisfaction with the cooperative’s governance and the perceived integrity and utility of the AI infrastructure, while supporting exceptional engagement with technology innovation.

**Conclusions:**

READ-COOP demonstrates that a cooperative business model has potential to sustain and support innovation in AI and ML infrastructures while promoting democratic participation and equitable ownership in particular contexts, in this case the cultural heritage sector. We suggest that cooperative frameworks are particularly suitable for AI infrastructures initially funded through public grants, providing a sustainable transition from public development to long-term, sustainable community-ownership, where focussed tool providers have adequate community support. We recommend wider application and exploration of cooperative models for innovation in AI and ML technologies for responsible creation, governance, and use, although we recognise READ-COOP’s unique context, community, and success may be an outlier.

## Introduction

Computational systems designed to process and search images of historical documents using Machine Learning (ML) based Automated Text Recognition (ATR) including Handwritten Text Recognition (HTR) now produce results of high accuracy. Yet implementing a bespoke system is fraught with difficulties, particularly for users in the cultural and heritage sector, such as historians, librarians, and linguists, who want to generate accurate transcription of historical documents without necessarily programming up the solution themselves. Since 2015 the HTR platform, Transkribus ( https://transkribus.org), has been offering a complete ingest, processing, and output service to generate high quality, accurate transcriptions from digital images of historical text, from any period or language. The first-to-market advantage has contributed to widespread uptake, with the resulting transcriptions underpinning thousands of research projects and hundreds of published papers, across a sweeping range of domains ( Nockels *et al.*, 2022). However, at the end of its European Commission funded development phase in 2019, the consortium of universities which developed Transkribus had to find a way to sustain the infrastructure and its community, while developing the platform and its Artificial Intelligence tools further. The solution was to found READ-COOP as a European Cooperative Society (SCE, *societas cooperativa Europaea*): an independent legal entity with the objective of fulfilling the needs of its Members, where common goals are shared and all earnings are directly reinvested to improve the infrastructure and its service. While platform cooperatives are not known for undertaking technological innovation, the establishment of READ-COOP effectively socialises the focussed product it delivers, providing institutional alliances, and pooling technical expertise. READ-COOP was officially established on 1 ^st^ July, 2019, and in doing so, became the first platform cooperative developing and hosting its own Artificial Intelligence and Machine Learning tools (the technologies which emulate human problem-solving capabilities).

We argue that the success of READ-COOP provides a blue-print for the establishment of other responsible and trustworthy AI cooperatives, where they could offer focussed technological platforms responding directly to specific sectoral needs, while undertaking innovation tailored to a particular context and community. Using a Reflection-In-Action methodology, this paper documents the development of Transkribus in the five years post research funding, continuing from the reporting in Muehlberger *et al.* (2019), providing a record of the establishment of READ-COOP, as well as detailing Transkribus’ technical developments. In addition, a survey of the Members of READ-COOP enables discussion of how the cooperative supports the delivery of the platform, and the potential benefits that have been provided by maintaining and innovating an Artificial Intelligence infrastructure under shared ownership.

We argue that cooperatives are an underused legal and business framework that can provide opportunities for technological developments to ensure that they are more responsive to a defined community’s requirements, and that they support accountability, as well as ensuring community engagement, which further supports sustainability, and the socialisation of technology platforms. We acknowledge that READ-COOP is highly unusual as a cooperative, having benefited from major EU grant funding and university facilities in its set-up phase. It also maintains a relatively niche technical infrastructure which is most used by researchers and institutions aligned to the specific library, archive, museum and wider cultural sector, benefiting from a first mover advantage and network effects in this space. Technical research and development activities that emanate from cooperatives have historically been rare, due to funding constraints and lack of access to knowledge and infrastructures. However, READ-COOP provides an exemplar which may be of use both to other technical providers, and researchers considering appropriate models to support the spin-out of digital platforms, as it represents a move away from the exploitative, extractive financial models that underpin much of modern digital platform infrastructure. The success of READ-COOP indicates that a needed digital tool, originally supported by grant income and university support structures in the research and development phase, can find its independence and financial supporters, provided it is embedded within its own context.

We argue that engaging in ethical business practices (including recognising the not-insignificant investment of public funds in the start-up phase) encourages sustainability of digital platforms, while ensuring a defined, and engaged, user community. We suggest that funders and technology incubators should support researchers in exploring cooperative approaches to technology spin-out and commercialisation, while also providing recommendations regarding the type of community, application, and activities which are most suited to cooperative AI governance: those which host products successfully responding to particular sectoral needs, with a large enough potential community to support activities. We also suggest that, when carefully supported, cooperative practices support engagement in innovation, allowing communities to interact with platform providers to develop tools to best support their needs.

Throughout this paper we refer to READ-COOP as the cooperative legal entity and employed delivery team which provides Transkribus, the information extraction platform. Members of READ-COOP are institutions and individuals who have purchased shares in the cooperative. Users are all individuals who have created a user account for Transkribus, which includes cooperative Members and non-Members. We also refer to the wider cultural heritage community which encompasses Members, Users, and non-users.

## 1. Methods

In order to capture and document the development of READ-COOP, and consider the effectiveness of cooperative AI infrastructure, we use a case study approach choosing an example of what is to be studied as a bounded system ( Flyvbjerg, 2011, 301): READ-COOP is the only platform cooperative we have identified that has developed and hosted their own AI and Machine Learning tools and is thus deserving of such attention. This, combined with Reflection-in-Action ( Schon, 1983), allowed us to identify “features of the practice situation – complexity, uncertainty, instability, uniqueness and value conflict” ( Schon, 1983, 18). Practically, an Action Research recursive methodology was used, where “continuing cycles of investigation designed to reveal effective solutions to issues and problems experienced in specific situations and localised settings, providing the means by which… organizations… may increase the effectiveness and efficiency of their work” ( Stringer, 2013, 1) via a focussed process of inquiry that allows the development of explanations that lead to increasing understanding ( Stringer, 2013, 5). This overlapped with collaborative self-ethnography approaches, where more than one researcher is involved in observing the interactions and behaviours of a group they are immersed in, using mixed methods “rather than just observation; participant observation… interviews, and other context-specific methods” ( Clapp, 2017, 34). In practical terms, this led us to consider: minutes from READ-COOP Board meetings; formal and informal evaluation reports; records of weekly team meetings and monthly Members’ meetings; posts to email lists and READ-COOP Slack Channel; and server reports regarding operation and usage of the system. Analysis was undertaken through regular team discussions on structured topics over a six-month period, with shared note-taking, interspersed with recursive co-creation, writing, and editing, setting aside periods of time to recover or locate dispersed information that had never been brought together, including using the Internet Archive ( https://archive.org) (indicating the ephemeral nature of records covering routine business activity ( Niu, 2025)). Complementing this, a survey of READ-COOP Members was undertaken, primarily to gauge their views of how READ-COOP was functioning, but also to consider wider aspects about the provision and sustainability of cooperative digital platforms. The survey was live between 21 ^st^ September and 21 ^st^ October 2023, hosted on Jisc Online Surveys ( https://www.onlinesurveys.ac.uk), a GDPR (General Data Protection Regulation) compliant research questionnaire platform. The survey was only shared with READ-COOP Members, via email and Slack channels. We did not target a wider online audience, preferring to reflect on active membership of READ-COOP. The study did not store personal information or data related to protected characteristics, and presented results are fully anonymised, with consent given by participants for publication of their comments. These activities facilitated the development and identification of operational themes ( Schon, 1983: 319; Stringer, 2013: 142). Research Ethics approval was given for this activity and method from the University of Edinburgh, as well as READ-COOP Board.

## 2. ATR, Transkribus and the READ project

Automated Text Recognition (ATR) ^1^ uses Machine Learning (ML) to generate accurate transcriptions from both print and handwritten mass digitised historical documents. This creates machine-processable text at scale, that can be then further searched, ingested, analysed or transformed, thus allowing libraries, archives, historians, and researchers greater, and more flexible, access to historic content. Since the mid 2010s, a range of software packages, tools, and infrastructures have existed in the ATR space ^2^, offering a variety of access to users and institutions, requiring differing levels of technical skills to implement or utilise them.

Transkribus ( https://www.transkribus.org) was the first handwritten text recognition (HTR) platform to provide access to ATR without requiring users to set up or manage their own machine learning or AI infrastructure, offering a complete ATR digitization and analysis pipeline without the need for technical training or knowledge of HTR implementation. This, combined with its effective user retention mechanisms and a network effect which encourages further adoption ( Belleflamme & Peitz, 2018), gained Transkribus significant “First-Mover Advantages” (FMA, see Kerin *et al.*, 1992; Papachristos & Suarez, 2022), and the platform remains the market leader in this space. It currently operates as a successful digital cultural heritage platform infrastructure, although offering a suite of tools in a relatively new domain. While publicly available HTR models enable users to recognize text within handwritten documents with no need for custom training, Transkribus Users can train their own HTR models on transformer-based neural networks (to further increase accuracy of particular hands or style of handwriting) and undertake trainable layout recognition (making it easier to process documents with complex layouts such as newspapers, periodicals, postcards, logbooks, legal records or forms). User transcription data is used to further generate and improve models to benefit other Users; this is done with full consent, and we openly communicate that data may be used in this way, to build and offer new services. Large-scale computing is necessary to support this data transformation (although the level of this depends on a variety of factors, including size of collection uploaded, and whether or not bespoke models are trained). The data and documents are stored centrally to build up a large data pool (READ-COOP has approximately 400TB of unique data, and 1.2PB of gross storage capacity including backups) which can be utilized for all machine learning ATR processes, for instance the training of general language models and improvement of publicly available ATR ( Colutto *et al.*, 2019). A range of other tools are now being developed by Transkribus, allowing Users to present, search, and comment on ATR results, with a vision to use AI to “unlock the written past” ( Transkribus, 2024c).

Transkribus outputs include transcripts, markup, and image segmentation, all exportable by Users. Users have full access to their data, which supports further research and development in an anonymized form. Collections, documents, semantics image segmentation, and ATR models can be shared, while core data infrastructure including our server components and ATR modules remains proprietary to support the business, as seen in Muehlberger *et al.* (2019). Users can become co-owners ("Members"), participating in meetings, communication, and decisions on technology development and commercialization. READ-COOP has faced criticism for not being fully "Open Source" or "Open Science" ( Vicente-Saez & Martinez-Fuentes, 2018) due to its proprietary core infrastructure. However, openly publishing Transkribus’ underlying model could lead to its misuse, threatening the READ-COOP business model and its community. Unlike major AI platforms, Transkribus prioritizes Member rights and transparent governance, aligning with FAIR data principles ( Wilkinson *et al.*, 2016) by being “as open as possible, as closed as necessary” ( European Commission, n.d.). This approach addresses constraints such as the (often illegal) scraping of cultural heritage content by large AI providers ( Brumfield, 2025; Bryant, 2025; Weinberg, 2025), and ensures balance between transparency and sustainability, protecting core systems for income generation, while Members enjoy significant rights.

READ-COOP ( https://readcoop.org), and its Transkribus platform, emerged from two distinctive European Commission funded projects. TranScriptorium (2013–15, €2.4m) ^3^, a consortium led by the Universitat Politècnica de València, produced the processing pipeline to generate accurate transcriptions of historical texts from digital images of manuscripts using Machine Learning ( Sánchez *et al.*, 2014). This resulted in the launch of the Transkribus Graphical User Interface: a downloadable Java-based client programme for local installation but connected to the central server, created by the Digitisation and Electronic Archiving group at the University of Innsbruck, in 2015 ^4^. The Recognition and Enrichment of Archival Documents (READ) project (2016–2019, €8.2m) ^5^, led by University of Innsbruck, focussed on further implementation and development of the user-facing service. By the end of the €10.6m funded period in 2019, a freely available, functioning platform containing a workflow for submitting and processing images was available, that was capable of a CER (Character Error Rate ^6^) of below 5% for handwritten text, and 1% for print material. This means that “AI is now enabling the near-perfect recognition of text from historical documents that were previously highly problematic” ( Neudecker, 2022, 1). Details of the development of the underpinning technology at this point, including history, workflow and use cases, can be found in Muehlberger *et al.* (2019). By early 2019, Transkribus was a functioning virtual research infrastructure, with 20,000 registered Users, and signed memoranda of understandings with over 60 institutions and projects, including leading libraries and archives ^7^. However, despite this success, the problem remained: how would it be possible to sustain the project and its platform after its grant funded period? The choice to establish the post-grant funded spin-out business as a cooperative, independent from the university context it emerged from, and the impact this has had on its development, Members, and Users, is the focus of this article.

## 3. Literature review: Business models for digital infrastructure

Maintaining and developing digital infrastructure is resource dependent, given platforms have continuous funding needs for maintenance, updating, and improvements, while managing technical challenges such as software obsolescence, data management, and cybersecurity, requiring a holistic view to be taken regarding digital sustainability ( Stuermer *et al.*, 2017). Any would-be-provider will have to grapple with issues of income, revenue, and profit to be able to undertake even core activities. When reaching the end of their European Commission grant-funded phase, the READ project had to consider a variety of options to sustain a viable ATR infrastructure. These decisions sit within a wider context of sustainability for cultural infrastructure, the business models that underpin digital platforms, cooperative approaches to business governance, and the aggressive capitalist approaches underpinning the rapid development of contemporary technology including AI.

### 3.1 The sustainability of digital cultural heritage infrastructure

In the Digital Humanities, a research methodology which foregrounds the use of digital technologies for the study of the Arts and Humanities, issues of sustainability, and lack of funding for supporting projects beyond their initial research grant-funded phase, are well known ( Barats *et al.*, 2020; Ross *et al.*, 2024; Tucker, 2022). There are, however, a few options for generating continuation funding. For example, consider the staffing and infrastructure of Transcribe Bentham ( https://blogs.ucl.ac.uk/transcribe-bentham/), the crowdsourcing project ( Terras, 2015) which encourages volunteers to read and transcribe the writings of the philosopher and reformer Jeremy Bentham (1748–1832) ( Causer & Terras, 2014). Transcribe Bentham provided the initial underlying training data for the Transkribus system. It continues to be supported by its host university department, UCL Faculty of Laws, following the completion of its varied grant funding (although this is a highly privileged and unusual arrangement given the current funding structures and financial constraints within Higher Education). A combination of institutional support and philanthropy can also support infrastructure, such as the Museum Data Service ( https://museumdata.uk) ( MDS, 2024). Crowdfunding is another potential funding mechanism which has had moderate success for resourcing non-commercial digital engagement projects in the Arts and Humanities ( Verhoeven *et al.*, 2013). An alternative is establishing a charity, with labour provided by volunteers and costs covered by donations and subscriptions: a route adopted by Programming Historian ( https://programminghistorian.org), which supports humanists in learning a wide range of digital tools.

Commercialisation is another approach for generating income, with universities now undergoing significant pressure to explore market opportunities and undertake entrepreneurial activities ( Compagnucci & Spigarelli, 2020). Unfortunately, while there are numerous commercial spin-outs that emerge from STEM (Science, Technology, Engineering, and Mathematics) disciplines, there are far fewer from SHAPE (Social Sciences, Humanities, and Arts for People and the Economy) fields ( Black, 2022), and many of these are social ventures (companies with a social or environmental goal ( Fellingham & Fraser-Walther, 2021)). In the Arts and Humanities, or the wider cultural and heritage sectors it is rare to make the journey from funded research project to self-sustaining, independent, commercial infrastructure.

### 3.2 Digital platform business models

There are a variety of potential income models that underpin digital infrastructures and allow continuation and development of service provision, and it is important to interrogate these, given limited resources ( Ross *et al.*, 2024). Models include: advertising, where advertisers pay based on engagement metrics; subscription-based models, where users pay recurrent fees; freemium models, where a basic level of provision is given for free while monetizing a subset of users who pay for enhanced service; surveillance and data analytics, selling user data to third parties for market research and targeted advertising; transaction-based models, which take a commission based on each successful trade; Platform as a Service (PaaS), offering tools to other providers to build on; crowdfunding; and partnering and sponsorship ( Kim, 2019). Additionally, income may be raised via: grants that support R&D and innovation via government or charities ( Jaskyte Bahr, 2019); venture funding or other forms of investment, where investors expect returns in the future ( Woolley, 2017); and token sales, where a digital asset such as cryptocurrency represents ownership, membership and voting rights (for example initial DEX or COIN offerings offered by Decentralized Autonomous Organizations (DAO) ( Rutskiy *et al.*, 2023)). A combination of these would be suitable for providing an ongoing income stream for the Transkribus service in its post-grant phase, particularly the subscription, freemium, and token model, but others, such as advertising and data analytics, were not appropriate for the product, its market, or its user base. There are sensitivities given the public funding which supported the development of the underpinning algorithms and datasets that drive Transkribus, as well as ethical concerns regarding the nature of the personal, historical data flowing through the system, and information about its Users, none of which is likely, nor appropriate, to be monetizable under surveillance capitalism ( Zuboff, 2019).

Most concerning is the nature of extractive commercial entities that deliver much large-scale technical infrastructure. Profit and growth motives drive late-stage capitalism: “there is one and only one social responsibility of business—to use its resources and engage in activities designed to increase its profits” ( Friedman, 1970, 126). Now prevalent “shareholder-oriented capitalism” ( Cheffins, 2021, 1607) means that large-scale technological providers are “… incentivized – and often obligated – to make whatever decisions will maximise shareholder profits without sharing those returns with workers or affected communities” ( MSI Integrity, 2020, p.8). This “is driven by the dual imperatives of digital capitalism: extracting data from, and expanding control over, potentially everything and everybody”, only advancing “the interests of corporate technocratic power… over other values like human autonomy, social goods, and democratic rights” leading to a “wide range of (un)intended and (un)known consequences” ( Sadowski, 2020, p.5). The social, political, and economic dimensions of data are controlled by ever-fewer monopolies, intent on utilising data for capital accumulation, domination, and extraction ( Huberman, 2022), with “our agency and autonomy becom[ing] further compromised” ( Scholz, 2023, 138), resulting in a “race to the bottom in terms of wages and working conditions” ( Schneider, 2021, 74). This has recently come to a head with the “near total ‘capture’ of AI research and deployment by corporations” whose “goal is to maximize corporate profit and to preserve (or even increase) the social and political power that enables it” ( Klein & D’Ignazio, 2024, 102) leading to “unequal, undemocratic, extractive, and exclusionary forces at work in AI research, development and deployment” (ibid, 100).

The business plans that underpin much of recent AI platform activity do not centre the sustainability or creativity of infrastructure, or the development of responsible AI (that which incorporates the benefits but mitigates the potential harms of AI, see Floridi *et al.*, 2018). Instead, General and Generative AI is “Extractive AI” ( Hogan & Lepage-Richer, 2024) which disrupts industries, markets, and employment practices ( Furman & Seamans, 2019), causes environmental devastation ( Hogan, 2024), and rides roughshod over intellectual property and human creativity ( Lee *et al.*, 2024), while concentrating wealth, resources, and power into a small technological monopoly which is increasing hard to dismantle ( Hogan & Lepage-Richer, 2024). While most major AI companies do have Responsible AI statements, these exist firmly within the capitalist framework outlined above and are therefore performative ( Kerr *et al.*, 2020) rather than engaging in any meaningful way with critical ethical aspects such as beneficence, non-maleficence, autonomy, justice, and explicability ( Floridi & Cowls, 2019). AI platforms tend to pursue monopolisation utilising shareholder value, lowering the efficiency of the market but concentrating their power, and acquiring innovative competitors. As a result, the end goal of most start-ups and spin outs in this space is the exit strategy – “the way funders and founders can cash out their investment…” but the focus upon this “leads to concentration in the tech industry, reinforcing the power of dominant firms. It short-circuits the development of truly disruptive new technologies that have historically displaced incumbents in innovative industries… the public loses access to many of the most promising new technologies… There has to be a better way.” ( Lemley & McReary, 2021, p.1). Instead, what would it mean to build a commercial digital infrastructure that prioritises sustainability, and keeping a service available for as long as possible? What would it mean to build an AI platform with and for and by a user community who were fiscally and intellectually invested in seeing it succeed?

### 3.3 Cooperative digital infrastructures

Cooperative infrastructures – jointly owned, democratically-controlled enterprises, by an autonomous community sharing economic, social, and cultural goals, with voting rights divided equally among members allowing them to engage with operational decisions ( ICA COOP, 2024) – are a modern type of business with a relatively long history dating back to the early 1840s, with many diverse forms ( Jones & Kalmi, 2013; Scholz, 2023). Cooperatives operate under seven key principles: Voluntary and Open Membership; Democratic Member Control; Member Economic Participation; Autonomy and Independence; Education, Training, and Information; Cooperation among Cooperatives; and Concern for Community ( ICA COOP, 2024). They are historically found in the agriculture, finance, housing, and education sectors ^8^. Coops have generally succeeded when they have mobilised community action to fill missing markets where there is a homogeneity of member interests ( Hansmann, 1999; Hueth, 2014). This shared vision and ownership emphasises cooperation and inclusivity, for the benefit of all members, and there is evidence that the democratic governance can contribute significantly to socioeconomic well-being, and support training and development ( Altman, 2009), thus advancing the UN’s Sustainability Goals for reducing inequality while spurring economic growth ( UN, 2023). Pérotin (2021, 18) discusses how worker cooperatives, by organizing production differently, tend to be more efficient than traditional firms, as evidenced by studies comparing their production functions. However, cooperatives have often been marginalised, and perceived as inefficient and ineffective, even though it has been shown that competitive marketplaces and cooperatives are not incompatible, and cooperatives have been resilient at times of economic crisis ( Altman, 2009).

There has been growing interest in cooperative ownership models for modern digital infrastructure ( Kelly, 2012; Scholz & Schneider, 2016; Scholz, 2023; Schneider, 2024), particularly in the delivery of microfinance and credit ( Kandie & Islam, 2022), and platform cooperatives that “introduce economic fairness, training, and democratic participation in the running of online businesses” ( Platform Cooperativism Cooperation, n.d.), provide shared community ownership for those selling goods and services in the digital economy. As of October 2024, The Platform Coop Directory lists 638 coops using digital infrastructure in 53 countries ( https://directory.platform.coop/), for example the Drivers Cooperative in New York City, supporting fair driver pay and working conditions ( https://drivers.coop). The distributed nature of platform coops may see “wider adoption and greater impact” than traditional brick and mortar cooperatives ( Scholz, 2023, 11) and the potential “to diversify the digital economy and to respond to the extreme lack of workplace democracy and the varying degrees of exploitation on digital platforms” ( Scholz, 2023, 6). This is not just altruism, but good business potential: research on start-ups shows that while, on average, only 44.1% of companies now survive their “difficult” first five years, “co-ops on the other hand have a survival rate of 80.4%” ( Glover, 2019). However, “the market share of actually existing platform cooperatives remains close to negligible” ( Schneider, 2021, 75. See also Schneider, 2018, 155–62). Most significantly, this is because of a lack of “start-up financing, which is already a major hurdle for traditional tech start-up entrepreneurs and becomes even more onerous for cooperative enterprises” ( Scholz, 2023, 12). Other issues include regulatory barriers, and the need for policy “to ensure that community ownership is at least as available to the online economy as investor ownership has been” ( Schneider, 2021, 76), as well as the need for barriers to be put in place “to constrain better-capitalized competitors that lack community accountability” ( Schneider, 2021, 78).

Technological innovation in cooperatives can be hampered by lack of access to finance, the lack of access to external knowledge, and lack of skilled personnel ( Basterretxea Markaida *et al.*, 2024). Despite these difficulties, recently some cooperatives have been established in the areas of development and hosting of software, data-led innovation, and data stewardship and governance. Cooperative ownership has been proposed as a new response “to the dilemmas of world-spanning digital platforms” giving opportunities for new efficiencies, improved internal regulation, and to prevent abuse of personal data ( Schneider, 2021, 74). The Platform Coop Directory lists 67 software companies ( https://directory.platform.coop/search/?s=Software). Many of these are building cooperative digital platforms for buying or selling services that bypass “Big Tech” providers, or groupware for collectives to support organisation, or provide access to worker collectives: see https://www.coops.tech for a further network of 37 cooperatives providing technology, digital, and creative services. Other data cooperatives deal with data stewardship, and open data services, enabling “the creation of open data and personal stores for mutual benefit” ( Mannan *et al.*, 2022, see also Mozilla Insights *et al.*, 2020) ^9^. The European Union’s Data Governance Act (DGA) has, from September 2023, enabled greater access to data, and “explicitly includes services provided by data cooperatives” expecting them to “help user-members make informed choices” ( Mannan *et al.*, 2022, 13–14), although the Act has been criticised for mentioning this “in passing” being described as a “missed opportunity to establish data governance mechanisms that reflect a ‘commons approach’ to data governance” ( Open Future, 2021), with Vogelezang and Keller (2021) noting “This concept should be better developed as it bears potential for users’ empowerment in the online sphere”.

In tandem to this, there are a growing number of blockchain-based Decentralized Autonomous Organizations (DAOs) that do not rely on a central authority for trust but facilitate “the creation of decentralized communities where participants have more direct control over decision-making processes” which “allow their members to control their own systems, made possible through the use of apps, dedicated digital assets, and rules they set themselves”, which are aligned to the principles of cooperatives, although further research is needed to understand how this recent type of democratisation and governance works in practice ( Peña Calvin, 2024). DAOs “circle of innovations and crises… presents an opportunity for reimagining networks along more democratic lines” ( Schneider, 2024 p. 13). However DAOs also have a questionable environmental footprint given the “energy consumption in blockchain networks” which “remains a critical consideration in assessing the overall sustainability of decentralized systems” ( Ekwere, 2024): a factor which is at odds with the cooperative principle showing concern for community. Nevertheless, these different discussions and proposals for cooperative approaches to digital infrastructures indicate that concept deserves further scrutiny.

### 3.4 Cooperatives and AI

As with the rest of the online environment, Artificial Intelligence and Machine Learning is becoming part of the cooperative digital landscape. Some digital service providers are worker coops that provide access to machine learning expertise (for example https://www.animorph.coop, https://code-operative.co.uk, and https://fiqus.coop/en/). Other data cooperatives may use AI or Machine Learning tools and libraries as part of their offering, particularly for data analysis, although it often remains unclear to what level they are engaging with, developing, scripting, or hosting AI themselves ^10^. Although data cooperatives have emerged “as a cooperative format, there appears to be relatively less emphasis on AI within the cooperative movement” ( Siebert, 2023). After a year-long search, we have found no other legally instantiated cooperative other than READ-COOP that openly develops, hosts, and promotes their own Artificial Intelligence or Machine Learning based platform to be used by others.

It is at this point that the reader may think of OpenAI ( https://openai.com), the sector leading AI provider of ChatGPT, the Large Language Model based chatbot launched on November 30 ^th^, 2022. OpenAI was founded in 2015 with the goal of developing artificial general intelligence which “benefits all humanity” claiming to

“research generative models and how to align them with human values” being “governed by a nonprofit and our unique capped-profit model drives our commitment to safety. This means that as AI becomes more powerful, we can redistribute profits from our work to maximize the social and economic benefits of AI technology.” ( OpenAI, 2024).

These statements reframe familiar approaches from the cooperative playbook. However, although it was established as a nonprofit in 2015, in 2019 when needing to attract investment “OpenAI came up with a bizarre hack. It would remain a nonprofit… But it would also create a for-profit entity… Behold a company that, depending on your time-space point of view, is for-profit and nonprofit” ( Levy, 2023, 73). Between 2015 and 2024, OpenAI raised $17.9bn in investment ( Tracxn, 2024). However, in September 2024 it announced plans to overhaul its corporate structure and become a for-profit business ( Milmo, 2024). OpenAI is therefore one of the legion of AI firms disingenuously co-opting the language of the cooperative movement for profit, demonstrated by this alleged corporate messaging bait and switch. Companies allegedly do this to appear as if they are holding human cooperation at their core, while concentrating power by exploiting profit-hungry investors, and maintaining a monopoly while funnelling profits to shareholders. This is a type of alleged commercial activity that Muldoon calls

‘community washing’ – the corporate marketing strategy of framing the activities of the company in the language of community empowerment and fulfilling a social mission. The aim is to drown out questions of substantial user fees, tax avoidance, venture capital funding and the company’s litigious history with stories… ( Muldoon, 2022, 50).

Other venture-capitalist backed AI companies allegedly playing this “co-opt co-ops” game include the for-profit Humane ( https://humane.com): “Technology that improves the human experience and is born from good intentions. Products that put us back in touch with ourselves, each other, and the world around us” ( Humane, 2024). We notice how attractive the fundamental cooperative operating principles are in AI marketing, and we encourage criticality to distinguish these traditional companies driven by monopoly visions and profit-making shareholder imperatives from those which are legally established as cooperatives and fully operating under cooperative principles.

It is useful to consider why cooperative AI infrastructures are rare. Operating in the AI development space remains resource-intensive because of the cost of necessary computing and access to expertise: start-up costs must be covered before profits are made, whether through philanthropy, investment, or donation. Unlocking start-up funding for AI software development remains a significant barrier to establishing any type of commercial entity. This is currently coupled with a speculative rush to own potential future revenues in this space, and a hype cycle defensively using the cost of training ever larger general LLMs to shield flaws while blocking competition ^11^. These hurdles mean, despite a few exceptions ^12^, that AI is currently

concentrated in the hands of a very small number of companies with outsized access to compute resources and diminishing transparency. Without urgent action, the benefits that [it] can bring to the world may be equally concentrated ( Nomic, 2023).

Core literature on platform cooperatives ( Muldoon, 2022; Scholz, 2023) does not engage meaningfully with AI development, and researchers have not yet published on how AI fits alongside the cooperative model, although Scholz’s earlier work recognised that “design for tomorrow’s labor market” will have to contend with AI ( Scholz, 2016, 26), and Camargo Benavides and Ehrenhard (2021, 974) call for further study of cooperative enterprise in innovation, including AI. This lack of consideration of AI cooperatives may be because of the prohibitive start-up costs of AI infrastructures which sees few companies at all operating in this space, the lack of understanding of cooperative frameworks as even an option for businesses ( Thorpe, 2019), or the relatively recent 2022 digital turn towards generative AI following the successful launch of the likes of ChatGPT which means AI cooperatives are still in gestation and will perhaps (or hopefully?) follow, meaning there are few available to discuss.

Yet the cooperative principles have much to offer particularly in the development of “responsible” AI: that which is developed and used in a legal way, without causing harm on perpetuating bias ( Floridi *et al.*, 2018). In their 2019 study, Floridi and Cowls distil six of the highest-profile sets of ethical frameworks for AI ^13^ into five overarching principles underpinning a unified framework for the responsible use of AI in Society: Beneficence (Promoting well-being of humans and sustainability for the planet); Non-Maleficence (Ensuring AI systems do not harm and promote privacy and security); Autonomy (Balancing human decision-making power with the growing autonomy of AI); Justice (Ensuring fairness, eliminating discrimination, and promoting equal benefit); and Explicability (Making AI systems transparent and understandable to ensure accountability). These can be mapped directly only the 7 cooperative principles ( ICA, 2024), as in Table 1, demonstrating the synergies between the two frameworks.

**Table 1.** The 7 Cooperative Principles ( ICA, 2024) mapped onto Floridi and Cowls (2019) 5 principles of AI in society.

Cooperative Principle	Map to AI in Society Principles	Explanation
**Voluntary and Open** ** Membership**	Justice	Justice, which emphasizes fairness and elimination of discrimination, aligns with open membership without discrimination and democratic approach.
**Democratic Member** ** Control**	Non-Maleficence	Non-Maleficence in AI promotes system security and privacy, aligning with cooperative values of accountability to members and maintaining control.
**Member Economic** ** Participation**	None specifically mentioned	The AI ethics principles do not directly address economic participation or financial management aspects typical of cooperative principles, such as reinvesting profits.
**Autonomy and ** **Independence**	Autonomy	The principle of autonomy, which underscores balanced decision- making between humans and AI, maps directly to cooperatives’ emphasis on self-rule and member independence.
**Education, Training, ** **and Information**	Explicability	Explicability in AI involves making systems transparent and understandable, which enhances user and developer knowledge, paralleling cooperative education goals.
**Cooperation among ** **Cooperatives**	None specifically mentioned	The AI ethics principles do not explicitly cover collaboration or solidarity among providers, technologies or their users.
**Concern for Community**	Beneficence	Beneficence, focused on well-being and sustainability for AI, maps directly to cooperatives’ goal of community-oriented sustainable development.

This mapping both indicates the suitability of cooperative principles for responsible and transparent AI governance and operationalisation, while also demonstrating two aspects missing from high level principles of responsible AI ^14^: appropriate management, governance and sharing of financial assets; and cooperation, collaboration, or solidarity between systems, technologies, or user bases. The cooperative principles are therefore a useful lens through which to consider the governance and organisation of responsibility and trustworthy AI, and we suggest responsible AI frameworks could be further expanded to encompass financial and community aspects ^15^.

Despite the attractiveness of cooperative language as a marketing tool for profit-driven AI companies, and the attractiveness of the cooperative model for the development of responsible and transparent digital platforms, we only know of one platform cooperative at time of writing who has developed and hosts their own AI and Machine Learning tools: READ-COOP ( https://readcoop.eu/), which is thus worthy of study and documentation.

## 5. Case study: READ-COOP

Transkribus ( https://www.transkribus.org) is now managed by the independent READ-COOP ( https://readcoop.eu) but emerged from an earlier set of EU funded projects and university-led collaborations. Reviewing the growth and development of the platform, its community, and its cooperative can also reveal how important the cooperative structure has been to the infrastructure’s success.

### 5.1 Origins: TranScriptorium and the READ Project

Transkribus was conceived and launched in February 2015 with funding from the European Commission’s Seventh Framework Programme (FP7-ICT ^16^), as part of the TranScriptorium project (2013–2015), led by Universitate Politecnica de Valencia ( EC, 2015), which focussed on the computational research necessary for accurate Handwritten Text Recognition (HTR). A successor European Commission Horizon 2020 funded project ^17^, READ (2016–2019), led by the University of Innsbruck, aimed to maintain, develop, and promote a functioning online research infrastructure where new technologies could feed innovation in archival research ( EC, 2016).

**Figure 1.**
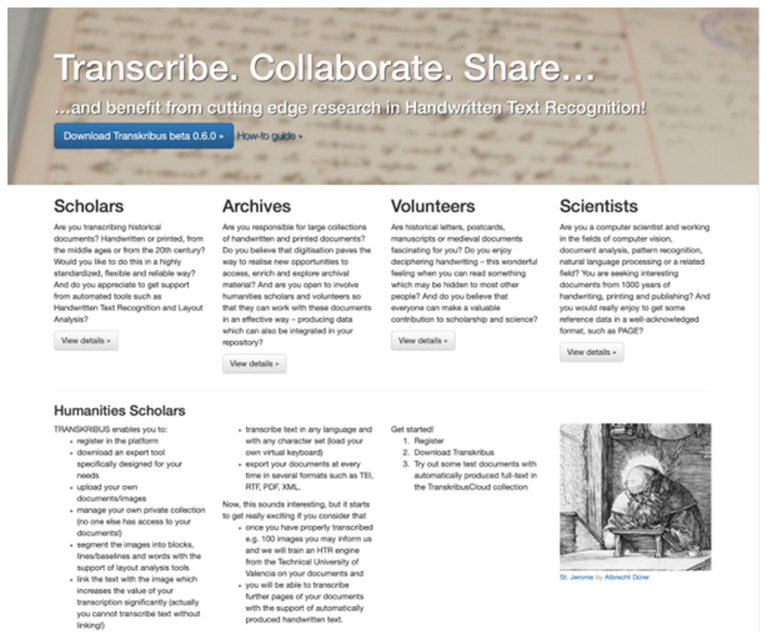
The original Transkribus website ( https://transkribus.eu/Transkribus/), at June 2015. Available from https://web.archive.org/web/20150606154718/https://transkribus.eu/Transkribus/.

**Figure 2.**
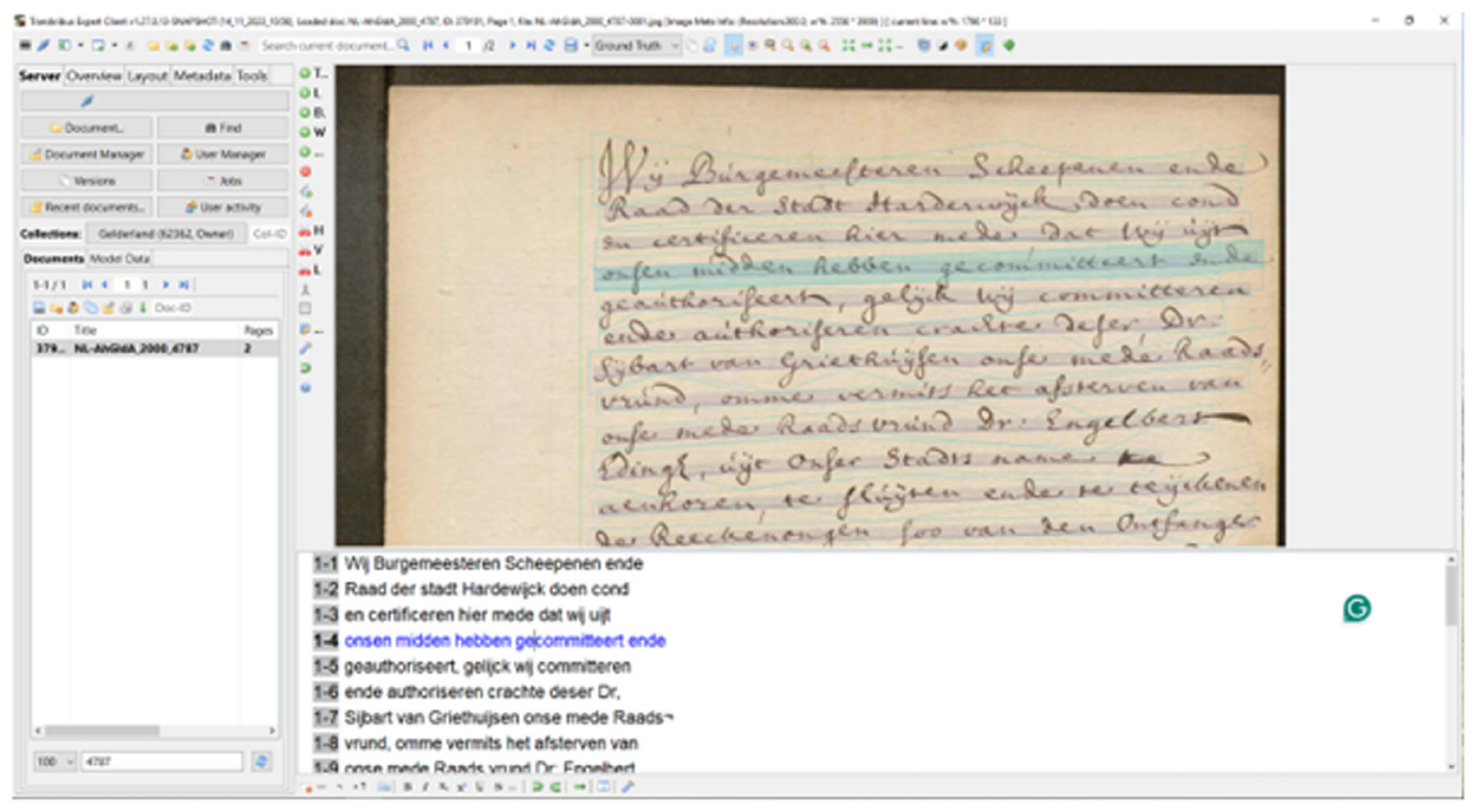
Screenshot of the downloadable Transkribus eXpert client (the Transkribus interface 2017–2023).

A recursive development and testing phase then ensued to develop its HTR, with a variety of heritage partners, researchers, Users, and tools development teams which were part of the READ programme ( Perdiki, 2019; READ-COOP, 2023a). The Transkribus GUI (Graphical User Interface) stabilised with the early 2017 release of version 1.0 of the downloadable desktop “eXpert Client” (see Figure 1), written in Java and C++ alongside OpenCV and JV ( Kahle *et al.*, 2017). This was the main way Users interacted with Transkribus to undertake transcription from 2017, until the launch of the web client in 2021, and the depreciation of the eXpert client in August 2023 ( Transkribus, 2023c). Users upload image files of manuscript material, with text baselines automatically detected. An annotation tool, and user-friendly interface, allows correcting of automated transcription, which can lead to training of bespoke HTR models, if necessary (see Figure 2) ^18^.

Between 2017 and 2019, continual development and integration of new advances in HTR occurred in the platform, such as the 2018 integration of Key Word Spotting and Apache Solr indexing solution enabling full-text search, which saw average Character Error Rates (CER) for handwritten material fall from 12% to 6%, and in some cases achieve a CER of 5% ( READ-COOP, 2023a). The development and operation of Transkribus software by the Recognition and Enrichment of Archival Documents (READ) project is fully documented in Muehlberger *et al.* (2019), with a more technical overview given in Colutto *et al.* (2019), and a detailed development timeline is also available ( READ-COOP, 2023a).

The Transkribus user community developed throughout the READ project coalesced around online fora, and in person user conferences held in Vienna in 2017 and 2018. By November 2018 the still free-to-access Transkribus HTR platform had 15,000 Users, which grew to 20,000 by March 2019, with word of mouth of this spreading within the archival, library, and Digital Humanities community (See Perdiki (2019) for an overview of how the platform operated at this time, including various screenshots). By this point, the platform was processing 100,000 documents and registering a maximum of 300 new Users per week ( Colutto *et al.*, 2019, 464). Transkribus had also built up a large machine-learning model for HTR (the millions of human interactions in correcting HTR generate reliable training data to improve the system), which was becoming increasingly accurate, achieving an average CER of 5% in validation-set scores, and therefore could be usefully applied to a wide range of primary source materials, increasingly print as well as handwritten materials. Users were training more than 200 bespoke ATR models every month, further increasing the accuracy of the system (training models incurred a small cost, operating under a freemium model). These growing digital assets alone were unique: “We assume that one would need to invest at least €2–3m for generating these data from scratch. It is to our best knowledge already today the largest and broadest HTR data set for historical scripts worldwide” ( Colutto *et al.*, 2019, 464).

However, the grant funded period of READ (which had effectively given Transkribus start-up funding) ended on 30 ^th^ June 2019, meaning that a new entity needed to be established to continue access to the platform, steward its digital assets, and also to provide income to ensure its sustainability and fund its continued development. On 1st July 2019 the READ project turned into a European Cooperative Society (SCE) with limited liability, independent from the University of Innsbruck. Six years later, READ-COOP SCE remains the legal entity which maintains and further develops the Transkribus platform.

### 5.2 Establishing READ-COOP

The establishment of READ-COOP came about after years of planning and ideation by the Principal Investigator of READ, Dr Günter Mühlberger, lead of the Digitalisierung und Elektronische Archivierung (DEA) at the University of Innsbruck, Austria. In 2012–2013, Mühlberger had been discussing the possibility of bringing universities together in a cooperative to access cheaper Optical Character Recognition (OCR) at wholesale prices due to volume. Mühlberger and Andy Stauder, who was also working in the DEA, consulted with the cooperative counselling service of the Raiffeisen-Landesbank ^19^ Tirol, an Austrian grouping of cooperative banks based in Innsbruck ( https://www.raiffeisen.at/tirol/rlb/de/privatkunden.html), that provides support for others wishing to establish similarly structured businesses. Although the proposed OCR cooperative was not established, the seeds for READ-COOP were planted, first appearing in the READ project proposal submitted to the European Commission in January 2015. The Impact section of the 2015 proposal required a Draft Business Plan for post-funding continuation of activities, in which an independent organisation was proposed, to be established during the funded lifecycle of the project, as part of a work package (WP3 Network and Business Development). In this proposal, Mühlberger noted:

If we consider an independent organisation we can see various attempts made in the last years to establish independent organisations arising from EU funded research projects, especially in the digital library domain… The legal models used are foundations, registered associations, or limited liability companies.
Horizon 2020 calls for “New forms of innovation in the public sector, open government, business model innovation” ^20^, therefore we like to mention a governance model, which does currently play no role in the discussion, but has proved to be a highly successful model: **the cooperative**. With the concept of “Shareconomy” cooperatives are now discussed as an innovative business model which fits very well to some of the requirements of the new economy. And indeed, cooperatives offer a number of advantages compared to the legal entities mentioned above. Cooperatives are not geared toward maximizing profits, but focus on optimizing their service for the co-op members who are also shareholders of the co-op. However, the co-op acts in a commercial manner with its members, as its services are completely offset and subject to supply and demand. The members are by no means compelled to purchase a certain service from "their" co-op, provided this service can be bought cheaper on the market. In this way co-ops manage to find a balance between the effectiveness of a private company and the collaborative spirit among communities following the same mission.
Additional special features of co-ops include their direct democratic constitution as well as the legal coverage of a member in the event of bankruptcy. Austrian cooperative law, for instance, ensures that in the event of bankruptcy, no more than twice the amount of the purchased share certificate is owed. Also mentioned is that the share certificates in a cooperative, contrary to other legal forms, can never become an item of speculation as the sale can only ever occur based on the nominal value thereof, but not based on the actual value of a cooperative. It will be an important task in *WP3 Network and Business Development* to make deeper investigations into this matter and to prepare an informed decision. ( Muehlberger, 2015, 42–43).

Interest from the Transkribus community was strong. However, support from incubators supporting companies “spinning out” from academic research was less forthcoming, given the more established route to establishing Limited Liability Companies that usually incorporates the university as a shareholder: READ would be the first spin out company in Austria to be set up as a cooperative. Ongoing discussions with Daniel Wibmer from the Raiffeisen co-operative founding consultancy service, and Günter Scheide from the Transfer Office Science - Business - Society at the University of Innsbruck, resulted in a presentation at the READ project meeting at Obergurgl in 2018 where the first version of the statutes of READ-COOP ^21^ were considered by the community: a proposal for a European Cooperative Society.

A European Cooperative Society (SCE) ^22^ is not a “not for profit”. It is a company that is obliged to make a profit, operating freely on the market: however, it gives both individuals and public institutions the chance to become co-owner of a company which is doing a service that is interesting and useful for them. The SCE is a legal entity that can be established by 5 or more European citizens (or a combination of natural persons and legal entities) from more than one EU country to allow its members to carry out common activities with limited liability, with a principal objective to satisfy its member’s needs. The condition of the SCE for transnational links reflects those already underpinning Transkribus’ development, given the READ Horizon 2020 funded project included researchers from 16 universities in 8 European countries, with 60 Memorandums of Understanding signed with other universities and research groups: READ-COOP could build on this network.

An SCE requires a minimum capital requirement of €30,000 ( EC, 2011): the initial capital for READ-COOP came from the first Members' shares and income from large-scale processing projects which had been contracted during the READ project by Günter Mühlberger, including: processing mass digitised manuscripts from the National Archives of the Netherlands ( https://zenodo.org/records/6414086); transcribing Finnish court records for the National Archives of Finland ( https://tuomiokirjat.kansallisarkisto.fi); and the Greifswald project, providing full text searching of German legal documents from the Baltic sea region ( https://rechtsprechung-im-ostseeraum.archiv.uni-greifswald.de).

After months of research, negotiations with stakeholders, and administrative preparations including the gathering of letters of intent to become cooperative Members primarily from the institutions who had previously signed Memoranda of Understanding (MoUs) with Transkribus, the founding ceremony of READ-COOP SCE took place on 1st July, 2019. There were 12 original signatories with a range of private, institutional, and commercial Members, including Leopold Franzens Universität Innsbruck, Austria (where the READ project had been based); Universität Greifswald, Germany (Dirk Alvermann, Head of Archives at Greifswald was an early and vocal supporter); Innsbruck University Innovations, Austria; Naver Labs Europe, France (a computer vision company partners in the READ project); Günter Mühlberger, Austria (TranScriptorium co-investigator and READ project lead); and Melissa Terras, United Kingdom (who had been a co-investigator of TranScriptorium and READ). READ-COOP SCE with limited liability was formally established and registered in the Austrian company register on November 15 ^th^ 2019, with the identification number FN 520187g ( https://justizonline.gv.at/jop/service/fba/teilauszug/520187g). Ongoing support from Innsbruck University Innovations ( https://innovation-innsbruck.at), such as access to working space, and university technical infrastructure, was crucial in the first few months until income streams began to flow. By this point more than 30 institutions and private persons had formally joined the cooperative ( READ-COOP, 2019). The original membership base in Germany, Austria and the Netherlands began a combined network effect ( Belleflamme & Peitz, 2018), reinforcing user retention and adoption in this concentrated and focussed market.

### 5.3 READ-COOP Structure

READ-COOP’s organisational structure is set out in its statutes ( READ-COOP SCE, 2022). READ-COOP has a Board of two to five directors, appointed for a maximum of three years, who are responsible for managing the SCE, subject to the European Council regulation on European Cooperative Societies (Council Regulation (EC) No 1435/2003), Austrian cooperative and company law with respect to the preparation of its annual financial statements and management report, and subject to annual independent cooperative auditing. At least two individuals serving on the Board are employees. READ-COOP Board oversees business operations including arranging the General Meeting, the appointment of Executive Directors, and are involved in strategic decisions.

READ-COOP directly employs a range of individuals, including Co-Executive Directors, Research and Software Engineers, Marketing and Customer Success Consultants, Content Managers, and Administrative support ( https://readcoop.org/team). The team structure combines technical expertise with user- and market-oriented roles, reflecting dual attention to development of technologies and integration of user perspectives. Employees report to the Co-Executive Directors, who report on operations to the Board. There are weekly All-hands meetings where employees and board members discuss activities and an active private Slack channel. Complementing the All-hands meetings, a system of domain-specific meetings supports coordinated decision-making, information sharing, and planning across the organisation. Employees are encouraged to become Members of READ-COOP, although this is not mandatory.

Members of READ-COOP (those who join by purchasing a share of the cooperative and paying an annual fee) can be Ordinary Members (natural or legal entities eligible to use the cooperative’s services) or Investing Members (not eligible to use the services but interested in the cooperative's purpose, such as research institutions or public authorities). Members formally exercise their statutory management rights in votes on key decisions, including amendments to statutes and policies, and overseeing financial matters. General Meetings may be convened by the Board as an ordinary or extraordinary meeting as needed. A monthly Members’ meeting is held online, with various talks, updates, and discussion points. Newsletters are circulated regularly. Members are communicated with often on Slack, to encourage community, dialogue, and information exchange, as well as feature development, bug-reports, and reporting of any incidents on productive systems. Employees of READ-COOP are encouraged to engage in these online Member discussions, for example the very active “ask-product” channel, which has multiple daily interactions with employees and Members. A “Weekly Read” Transkribus news keeps staff and Members informed.

All Users of Transkribus (i.e. individuals who create user accounts to access and use Transkribus tools) are encouraged to attend the Transkribus User Conference (TUC), held in hybrid format approximately every eighteen months. The TUC reserves presentation slots for Early Career Researchers and low-income Users, who can gain supported access to processing via the Transkribus Scholarship Programme. Users are also contacted by a variety of digital methods including newsletters, and social media. Users are encouraged to become Members, but this is not mandatory. Users can interact with the READ-COOP team via help desk function but cannot access Members discussions and meetings.

### 5.4 READ-COOP growth 2019–24

Since the creation of READ-COOP SCE in 2019, Transkribus has continued to be developed, the cooperative membership has expanded, and registered Users of the software have increased. The number of pages processed continues to grow, and more new models are being developed, including those openly published to be reused by others. The revenue stream supporting Transkribus has diversified, to move from the freemium to software-as-a-service (SaaS) model ^23^ (still with a number of free credits to support trial and experimentation with the platform), and the platform also generates income from large scale ATR contracts. It is useful to look at the documentation surrounding this to understand the functioning and culture of the cooperative, including the growth of number of individuals employed to service the community, which also indicates READ-COOP’s positive economic impact, mostly in Austria.

**Figure 3.**
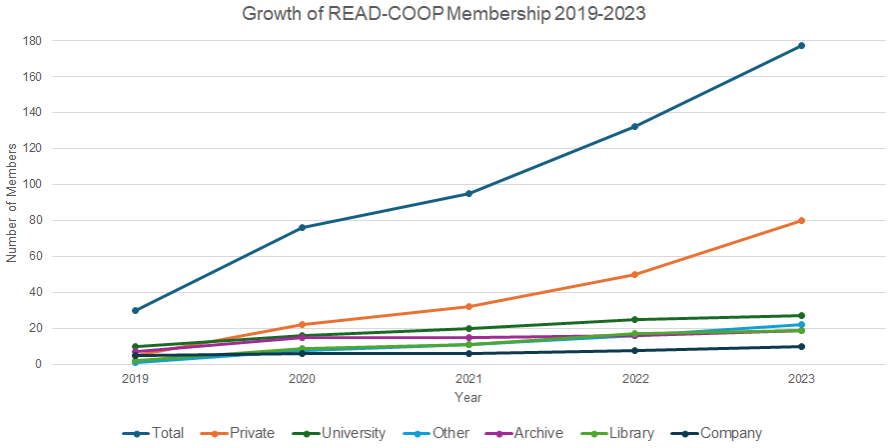
Growth of READ-COOP Membership from instantiating as an SCE in 2019.

***5.4.1 Membership.*** Growth in membership has been steady, showing the support for the Transkribus infrastructure, and willingness to be involved in the community (see Figure 3). Managing Director of READ-COOP, Andy Stauder, commented in 2021:

We knew that right from the start there would be some Members, because we had collected letters of intent, in order to have a somewhat safer ground on which to build the whole thing. But that it would become such a successful model and that we would make it across the pond, among other things - Stanford University recently made its commitment to membership - is something we never expected and that it would happen in such a short time. (Translated from the German, Transkribus, 2021a).

As of 11 ^th^ October 2024, there were 227 Members of READ-COOP. There are two distinct ways to become a Member. Individuals can join as private persons, for a membership fee of €250 which gives one share (individuals can have a maximum of 1 vote), with an annual individual membership fee of €62.50 (including VAT). Institutions can join by purchasing 4 shares at €250 each (which translates to one vote). The maximum number of votes that can be cast by an institution is 5, which can be reached by purchasing 20 shares. The number of shares an institution can purchase is unlimited (allowing them a vehicle to further invest in the infrastructure) but has no further impact on the number of votes available. There is also an annual institutional membership fee of €250. Further shares can be purchased by private and institutional Members and sold back to the cooperative, if necessary, without increasing their voting access beyond the maximum: this is a mechanism by which Members can financially support the liquidity of the cooperative. All Members access a variety of benefits including discounts on credits for processing documents on the system, as well as input into business policies ( https://readcoop.eu/join/).

As of October 2024, 45% of READ-COOP Members are private individuals (see Table 2) and growth of the private membership option is strongest over time, showing how invested individual Members have become in the operations of the cooperative, although overall voting share remains low. Over a third of memberships come from Universities, Libraries, and Archives, who own 56% of READ-COOP shares: we often see these joining to support a range of activity within their institutions, although growth in institutional Members is more gradual than the private category. “Other” is used to represent a range of other organisations including Museums, Academies of Science, Foundations, Societies, Institutes, Schools, Publishing Firms, and War Memorials, who own 24% of READ-COOP shares. There has recently been a growth in the number of companies becoming Members, who own the remaining 10% of READ-COOP shares, indicating the increasing embedding of pipeline and outputs into other commercial workflows. Since taking out membership, only 4 Members have cancelled (2 private individuals, and 2 companies): Member retention levels indicate engagement, with most remaining in the cooperative once they have joined.

**Table 2.** READ-Membership and share ownership by Institution Type, as of October 2024.

READ-COOP Membership By Type	Percentage of Total Members	Percentage of READ-COOP Share Ownership
Private	45%	10%
University	17%	19%
Other	12%	24%
Library	10%	21%
Archive	10%	16%
Company	6%	10%

The majority of Members are from Europe (85%). We have especially strong membership numbers in Germany (19%), Austria (14%), and the Netherlands (14%), reflecting the network of Universities, Libraries, and Archives built up during the H2020 READ project. 10% of READ-COOP Members are from North America, 2% from South America, 2% from the Middle East, and 1% from Australasia. We are actively reaching out to potential membership organisations beyond Europe, and understanding how we can support scholars, institutions, and organisations from the Global South, responding to calls from the Digital Humanities community for inclusive practice ( Risam, 2018). For instance, READ-COOP SCE were recently awarded a grant from the Bill and Melinda Gates Foundation ( https://www.gatesfoundation.org) to transcribe Nigerian healthcare records “and programmatic data that is still found in paper forms for greater visibility and use in decision making” ( Bill and Melinda Gates Foundation, 2024).

From these membership figures, it is evident that income from membership fees is not enough to support the Transkribus infrastructure alone. The platform moved from a freemium (free-to use generic ATR models/pay to apply bespoke ATR models) service to a credit-based model (still giving access to some free credits to allow trial and supporting hobby Users, but with a priced credit structure based on volume of pages to be processed) on 19 ^th^ October 2020 ( READ-COOP, 2020), with an updated pricing system (giving 100 free credits per month instead of 500 free credits in total) launched in January 2024 to bring more flexibility and increased value ( READ-COOP, 2023b). Initial free credits operate as a means for Users to trial the platform, and to be able to undertake the processing of small projects for free to allow as much access as possible, but are also a marketing tool ( Li, 2022). When combined with network effects and the closed nature of the software and models, the free credits also function as an effective user retention mechanism. Discussions with Members are key when developing and updating pricing plans and access models, to best reflect the needs of the membership community, while covering the costs of the infrastructure.

***5.4.2 Member Engagement in READ-COOP.*** Attendance at cooperative movement management meetings has been noted as being low in cooperatives across the world ( Mohd-Saleh *et al*., 2025), with previous research showing “no matter what size of region 95% or more of cooperative members decline to be involved in their Society’s governance” ( Davis & Donaldson, 2000, 160). READ-COOP records show a different story – with an average Member attendance of 42%, and average voting rate of 55% (some Members hold more than one vote), see Table 3. Attendance at informal monthly meetings was between 50 and 75 Members attending (out of a growing list of between 209–255) throughout 2024. This demonstrates exceptional success in engaging READ-COOP Members with READ-COOP management, business delivery, and innovation engagement – and it could be argued that the cooperative aspect of Transkribus is as much of a support for technological innovation as it is governance, although it can also be seen that as the number of Members expands, a smaller proportion is attending the AGM over time.

**Table 3.** Member Engagement and Voting in READ-COOP Annual General Meetings.

AGM Year	Total Members	Members Present (or by Proxy)	% Members Present	% Registered Votes Present
2020	55	34	61.8	81.9
2021	87	49	56.3	69.1
2022	113	41	36.3	53.6
2023	147	58	39.5	51.1
2024	209	61	29.2	36.4
2025	255	75	29.4	40.6
***Average***	***144***	***53***	***42***	***55***

**Figure 4.**
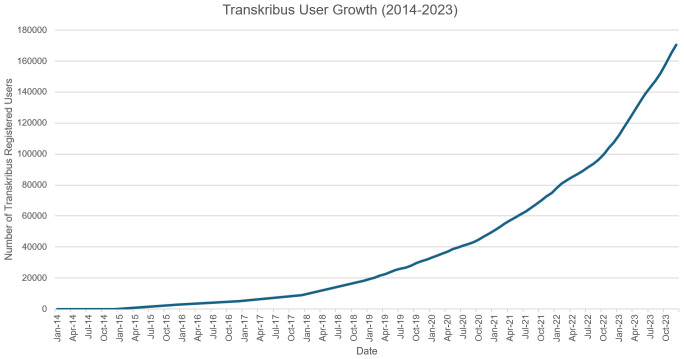
Cumulative sum of registered Users of Transkribus over time.

***5.4.3 User growth.*** User growth for Transkribus has been steadily increasing since 2019: at the launch of READ-COOP, there were 25909 registered Users, growing to 170643 by close of 2023 (see Figure 4). In early May 2024 the platform reached 200,000 user accounts ( Transkribus, 2024a), growing to 235,000 accounts by October. From user growth numbers in both the Users and Member graphs it can be noted that the switch to the credit-based software-as-a-service model in 2019 has not impacted use of or support for Transkribus (Users of Transkribus were surveyed before this change, with half of respondents concerned with the sustainability of the tool after its grant-funded phase ( Terras, 2022). A future User survey is planned).

**Figure 5.**
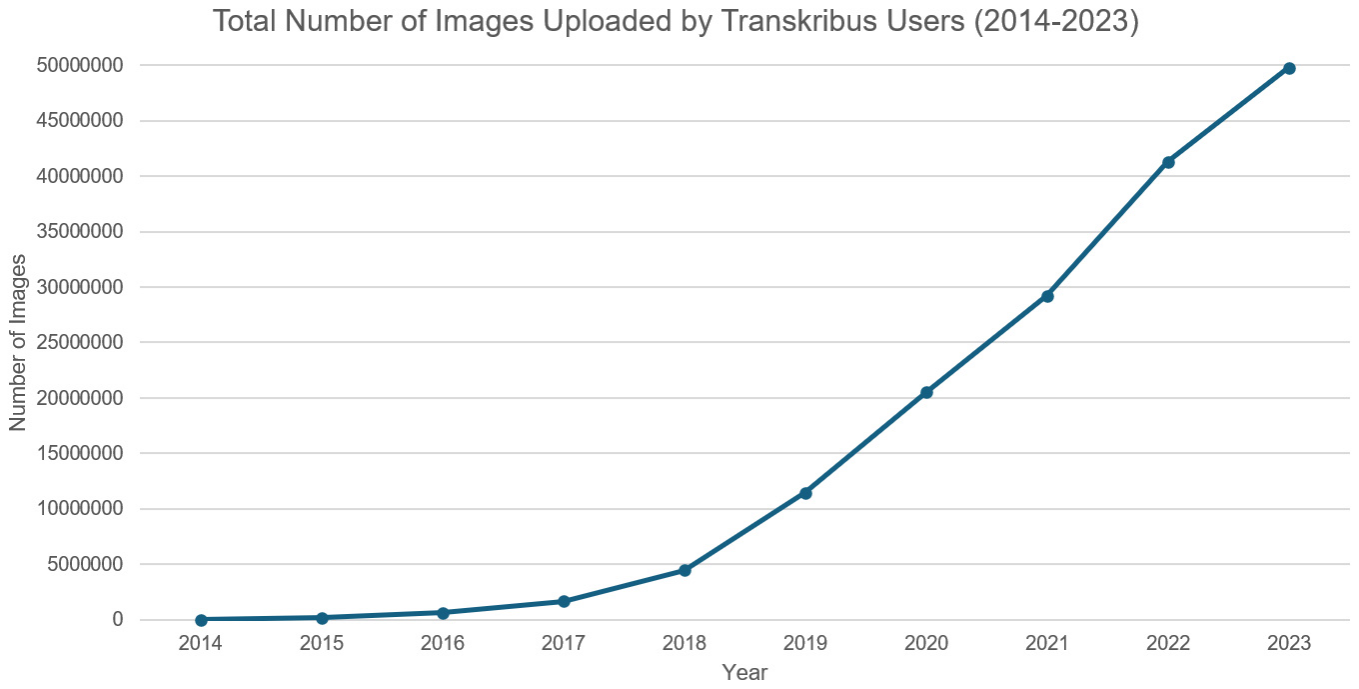
Total number of document images uploaded by Users onto READ-COOP servers over time.

***5.4.4 Documents processed.*** A total of 50m images were uploaded by users between the establishment of READ-COOP in 2019 and end of 2023 (see Figure 5), with over 15m additional images processed directly by READ-COOP on large-scale ATR contracts, delivering HTR transcription for a variety of external parties. By October 2024 READ-COOP infrastructure had processed over 90m individual images of pages of text, with user uploads now averaging 1m pages a month.

**Figure 6.**
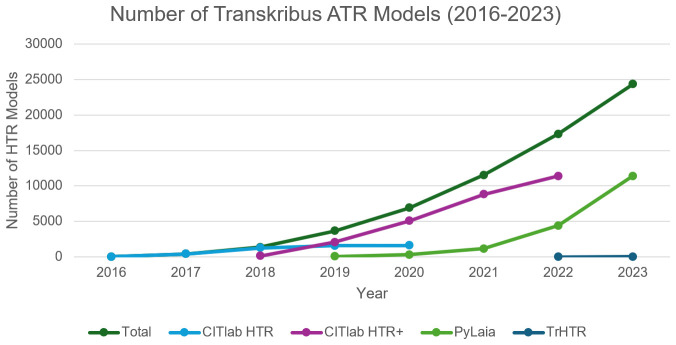
Number of ATR models generated by Transkribus, in total, and by integrated baselined recognition technology.

***5.4.5 ATR models created.*** A number of baseline recognition engines have been used in Transkribus (see Figure 6), including CITlab HTR and CITlab HTR+ (also known as HTR+, developed by the University of Rostock, see Strauß *et al.*, 2018. HTR+ was available in Transkribus until November 2022), and PyLaia (developed by the University of Valencia, see Sánchez *et al.*, 2019), both developed as part of the EU funded READ project. Since 2024 READ-COOP has its own Transformer HTR (TrHTR) baseline engine, adapted from TrOCR ( Li *et al.*, 2023). The growth of interest from Users in creating their own models is clear, and many are then made public for others to use ( https://readcoop.eu/transkribus/public-models/) or used to train further iterations of the system, as is the case with TrHTR (Transkribus READ-COOP, 2023j, see also 5.5 below).

***5.4.6 READ-COOP Workforce.*** Normal business operations of READ-COOP began in November 2019. The first employee of READ-COOP was Andy Stauder, Managing Director, who was also an early private Member of the cooperative. The growth in membership and document processing volumes over the past four years has necessitated a steadily growing workforce in READ-COOP (see Table 4), both to service existing use and infrastructure, and to promote and further develop Transkribus’ offer, which has been enabled by the profits of the company being reinvested into its infrastructure. The success of the business has made a notable contribution to the local economy. However, after a phase of rapid growth, the Transkribus workforce underwent a slight reduction in early 2024. This strategic adjustment lowered personnel costs from 85% to 70% of turnover, aligning with the cooperative's goal of ensuring long-term sustainability. As of October 2024 (time of writing) the headcount is 36 with 20.75 FTE.

**Table 4.** READ-COOP Workforce, at end of year.

**Year**	**Headcount** ** (31.12)**	**FTE (31.12)**	**FTE (avg/** **year)**	**Board** ** Members**
2020	3	3	2.55	3
2021	12	8.01	6.18	3
2022	29	16.84	13.88	3
2023	35	22.68	19.3	5

Seven employees (and two freelancers) are currently Members of READ-COOP (31% of current FTE). The growth in activities has also necessitated a recent growth in the number of board members for adequate oversight and governance. As of October 2024, two board members were employees (and Members), showing a mandatory direct link with the workforce in READ-COOP management structure, as set out in the cooperative statutes (Andy Stauder, Managing Director, and Florian Stauder, Employee Board Representative). Board members, and private Members, Günter Mühlberger (Chair), Melissa Terras (Scholarly Director) and Annemieke Romein (Community Director) do not draw salaries from READ-COOP ^24^.

### 5.5 Supporting READ-COOP culture

Building a successful cooperative requires building a strong culture, necessitating the “integration of shared rituals… and celebratory events as an integral part” of cooperative ethos ( Scholz, 2023, 19), and the need to build “a strong sense of community and identity, as well as a strong business venture” ( Scholz, 2023, 183). This is particularly the case given the number of staff working remotely, as well as the distributed membership base. READ-COOP has done this in a variety of ways, using digital, online, and hybrid approaches. As described above in Section 5.3 and Section 5.4.2, a dedicated Member-only Slack channel allows regular asynchronous communication with project staff and prioritised problem solving and technical support, monthly meetings are held, and Members are encouraged to attend our Annual General Meeting for formal auditing of cooperative business.

By doing so:

we heavily involve Members… we gather direct input on lots of things. So, there’s direct democracy built into the way we conduct business. E.g., we launched an information campaign about changing the pricing system, or moving from a java desktop version to the web, and collected and took to heart the Members’ feedback all the way ( Stauder, 2023).

It is then up to the Members to determine how much they wish to engage:

Of course, it varies somewhat how much the individual Members are involved in the cooperative, i.e., how much they participate in the democratic process, but there is a very high level of participation in the general meeting, for example. That means people are really interested in what happens in the cooperative. From that point of view, it really feels like a “co-op” from the inside as well (Stauder, Translated from the German, Transkribus, 2021a).

Beyond the immediate operations of the cooperative, Members and Users are encouraged to discuss their work and build their own network and “community of practice” ( Wenger, 2000). The in-person Transkribus User Conference was held in Vienna (2017, 2018) and Innsbruck (2020, 2022, and 2024), with papers being video-recorded, and broadcast online. This is also an important event for READ-COOP staff to get to know and interact with Users. Members of READ-COOP are also encouraged to attend disciplinary conferences and meetings and advocate for Transkribus, such as the meet up at the annual international Digital Humanities Conference 2023 in Graz ( Transkribus, 2023b). READ-COOP also supports training materials that are available to host workshops, events, and webinars, and support is available from READ-COOP staff.

Since October 2020 (coinciding with the move to a credit based system), a Transkribus Scholarship Programme has offered free credits to those hosting training events, and students can apply for free access to Transkribus systems ( https://help.transkribus.org/4.-transkribus-scholarship). By October 2024, 336 students from 73 countries (including individuals from Africa, South America, Asia, Middle East, and Oceania as well Europe and North America) have so far been supported in their studies, with READ-COOP providing over 1m free credits, showing that Transkribus is “bearing in mind user needs and the promotion of diverse research” while balancing this with financial sustainability of its tools via revenue generation from more financially established Users and institutions ( Nockels *et al.*, 2024).

**Figure 7.**
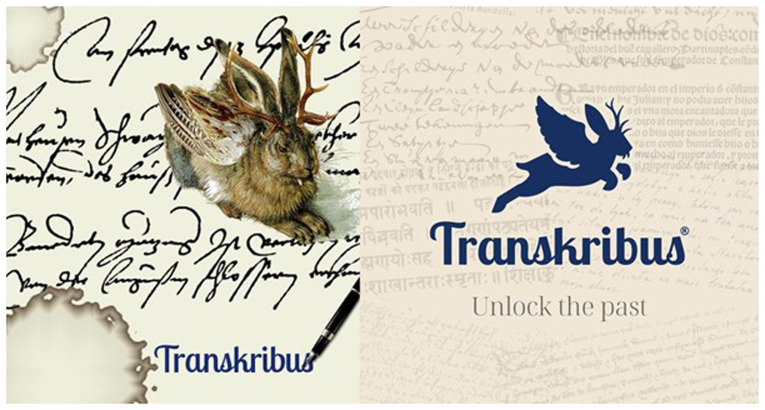
The evolution of the Transkribus brand: Dürer-based Wolpi (used 2015–2023), and new 2023 logo.

A robust social media presence supports all these activities, including a discussion forum on Facebook. Since 2015, and the launch of the READ project, Wolpertinger, a version of Albrecht Dürer’s 1502 watercolour “Young Hare” edited by an unknown artist, has been the mascot of Transkribus ( Transkribus, 2015). The Wolpertinger reflects the Bavarian-folklore heritage of its origins in Innsbruck, and (it has been argued) represents the heterogenous data inputs that Transkribus ensembles into a cohesive transcript ( Carlbla, 2020), in a tool that can do many different things. The mascot was nicknamed “Wolpi” by Florian Stauder in 2021 ( Stauder, 2021), and in January 2023 a new Transkribus logo icon incorporating a modern interpretation was unveiled (see Figure 7), after READ-COOP Member feedback and co-design ( Transkribus, 2023a). Wolpi now adorns merchandise including laptop stickers, t-shirts, and hoodies, further promoting Transkribus, assisting with story-telling and brand-recognition, and building a sense of community.

Members (and data gathered from Users, with consent) are also involved in tool development, where data-driven prioritization is now routinely utilised: Member interviews are used to build surveys to collect data over broader groups, to enable understanding of user needs. Mock-ups and wireframes of new features are also tested with Members in advance of programming, allowing efficient product design and rollout.

### 5.6 Technical advances in Transkribus since 1 ^st^ July 2019

**Figure 8.**
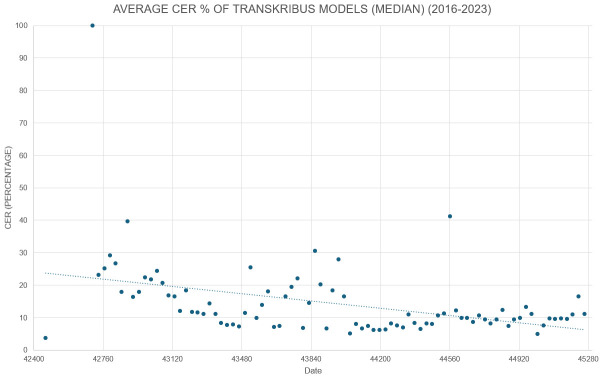
Average (Median) Character Error Rate (%) of all Transkribus HTR models over time.

Since its incorporation as a cooperative in 2019, the investment of profits into the infrastructure has allowed Transkribus to develop its technology, interfaces, and support structures, along the way making the transition from academic research software to fully fledged, commercial SaaS product. Sustaining the infrastructure (and its other products that link to it, such as the DocScan app (available for Android only, https://help.transkribus.org/docscan), and the ScanTent, with over 1000 units sold to institutions and individuals worldwide, ( https://www.transkribus.org/scantent) requires resource within itself. Continued engagement with Members allows Transkribus to function in a responsive and agile way, to develop and improve. This includes an overall increase in the accuracy of Transkribus’ ATR, which is indicated by a decreasing Character Error Rate in validation-set scores (see Figure 8), showing an overall trend improvement in functionality of the model. Retained records for CER begin with the CITlab HTR recognition engine created by the University of Rostock team as part of the READ project in 2016. The gradual improvement shown is not linear due to the introduction, testing, and refining of different ATR engines over time (CITlabPlus, Pylaia, TrHTR). The average CER for handwritten text is currently 5%.

There have been a variety of technical changes to the Transkribus platform since 2019, including: the shift to web-based tools, away from the eXpert client; the updating of the website, branding, and user support functions; the launch of a web-based publishing solution which enables Users to search collections historical manuscripts that have been transcribed with Transkribus; and the further development of ATR tools and models. During this time, transitioning out of the University of Innsbruck’s infrastructure — including office space, storage, database, and high-performance compute — was essential for the cooperative’s autonomy, but required substantial effort: the cooperative now operates entirely independently of the university setting it emerged from. It should also be stressed that since establishment, all electrical power for READ-COOP administrative activities and Transkribus computational processing comes entirely from 100% renewable resources, via TIWAG ( https://www.tiwag.at): this both recognises and ameliorates the environmental and societal harms encompassed by AI ( Lehuedé, 2024), while responding to the cooperative principle regarding caring for community.

**Figure 9.**
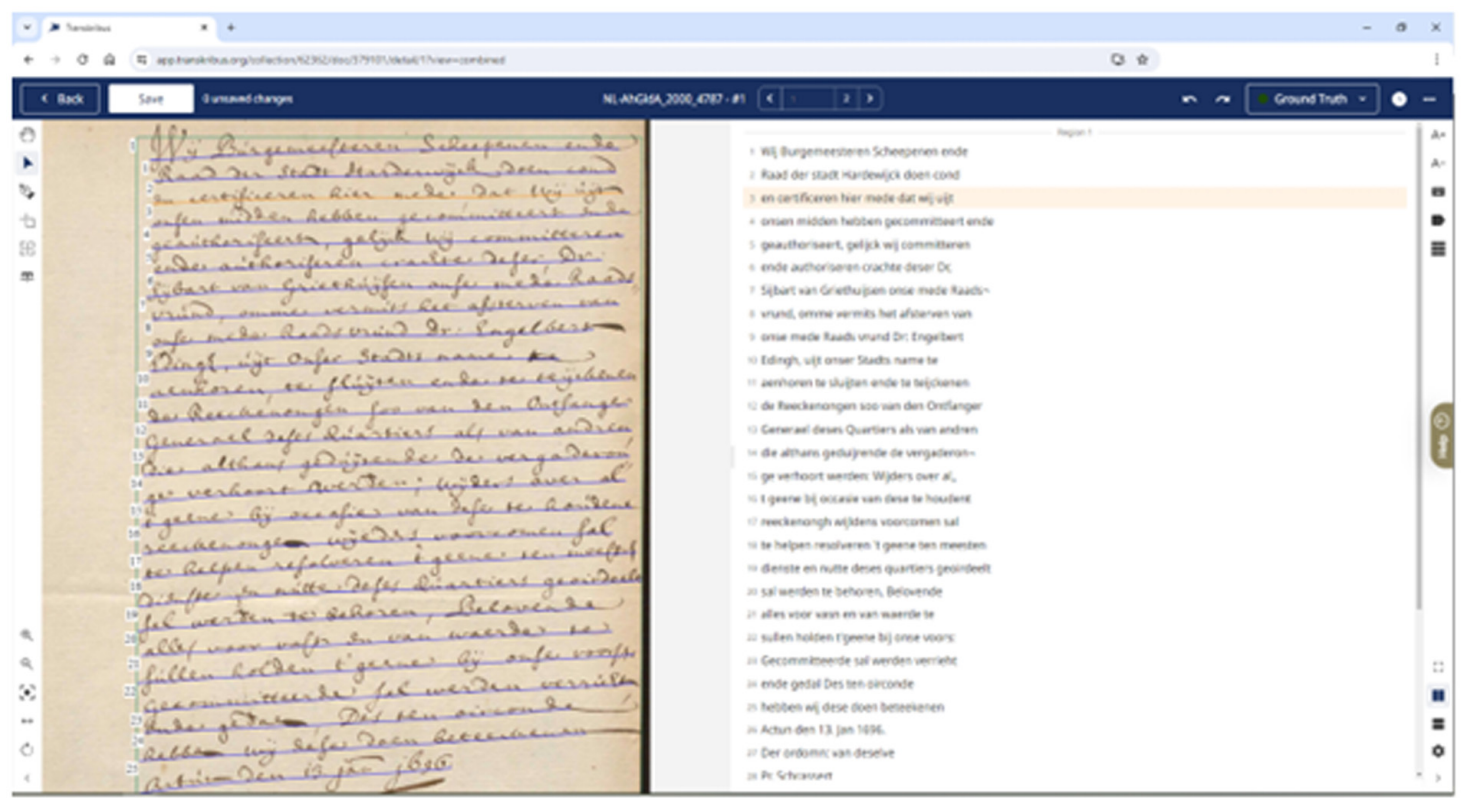
Transkribus web app, launched August 2023: the online version of Transkribus, accessible online ( https://app.transkribus.org/home).

Transkribus has recently undergone a shift to web-based tools, instead of continuing to host Transkribus via a downloadable eXpert client. This was necessary for a variety of reasons, including more efficient processing, and ensuring all Users have access to the most up to date tools with a consistent user experience across different operating systems and specifications, as well as allowing access to documents from computers and mobile devices. This has been a major work package for READ-COOP delivery team. Transkribus Lite launched in February 2021 ( READ-COOP, 2021a; Transkribus, 2021b), and functionality has been gradually added since. In August 2023, the new Transkribus web app was launched (replacing Lite), which continues to be developed ( Transkribus, 2023c), see Figure 9 ^25^. Users can upload image files of manuscript material to the web app, with text baselines automatically detected. An annotation tool, and user-friendly interface, allows correcting of automated transcription, which can lead to training of bespoke ATR models, if necessary. Since the launch, Users have been encouraged to port all activities from the eXpert client to the Transkribus web app. All features from the eXpert client are expected to be fully integrated into the web app by early 2025, and the eXpert client then withdrawn ( READ-COOP, 2023f). While this gradual shift of tools has brought some uncertainty to power Users – some features of the eXpert client are still being added to the web app – the upgrading as well as maintenance of infrastructure is crucial to the future success of the cooperative.

**Figure 10.**
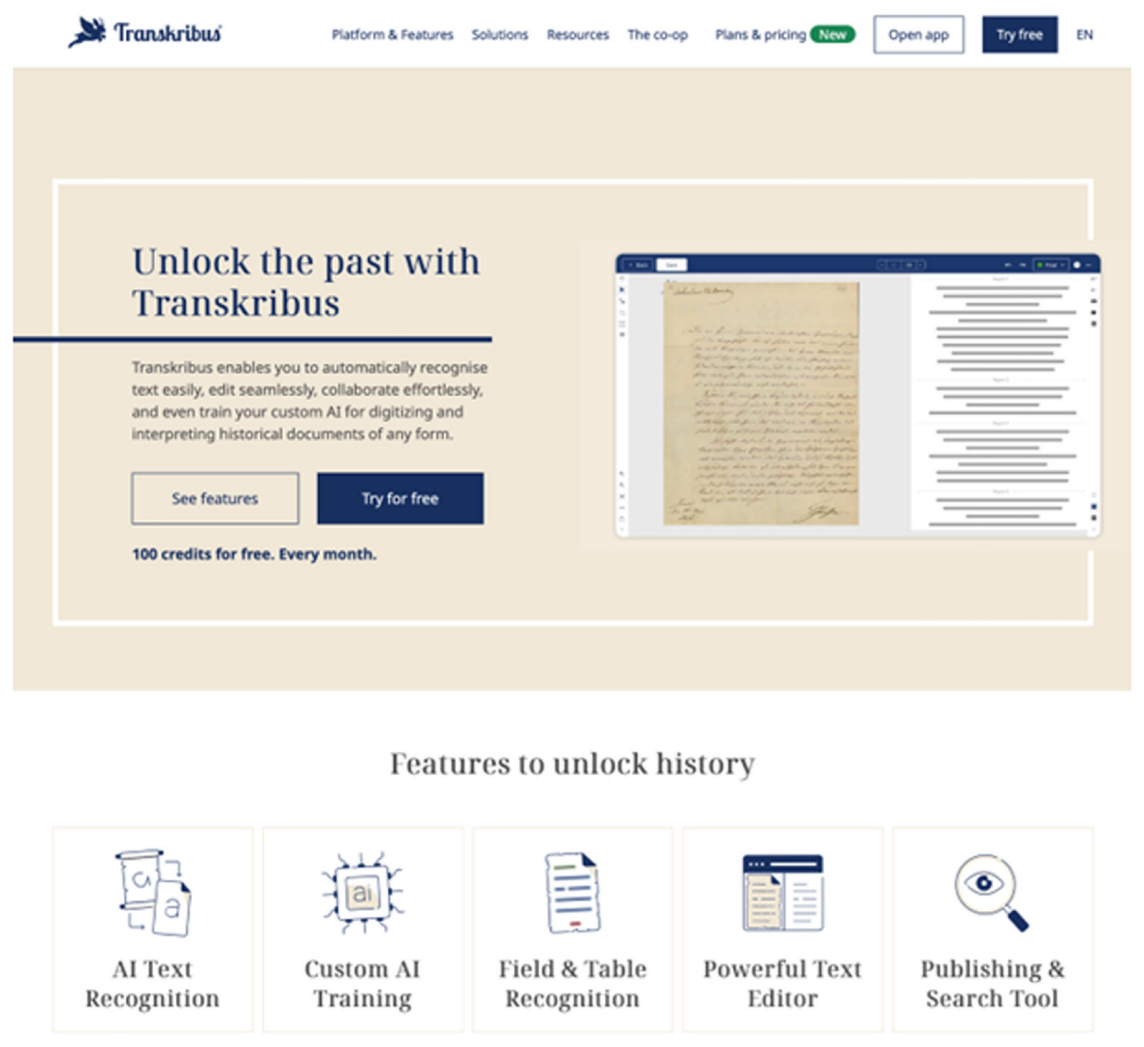
The streamlined 2023 Transkribus webpage with new, cleaner, design and branding.

The website itself has also had a major refresh and rebrand, integrating many other support mechanisms and tools, with improved accessibility. In 2021, the introduction of a free, web-based drag-and-drop ATR service for the general public ( https://transkribus.ai) has allowed access to and experimentation with the basic ATR process in a WYSIWYG (what-you-see-is-what-you-get) web environment: from July 2023 this has been embedded into the new https://www.transkribus.org homepage (see Figure 10) to allow prospective Users to try the service ( READ-COOP, 2023k). In addition, at the same time, a new help centre ( https://help.transkribus.com/) launched, making support routes clearer to the user community.

All material in Transkribus—including transcripts, in-document annotations, metadata, and document images — are fully exportable at any time in open formats (e.g. PAGE XML). Users are entirely free to build their own digital editions elsewhere, and many do. However, we noted many Transkribus Users, especially in academic and heritage contexts, struggle to host and present their material effectively — even after completing information extraction work. In 2021, Transkribus launched a web-based publishing and hosting solution, read&search ^26^. It allows collections of documents that had been processed within Transkribus to be shared with the general public or with logged-in Users via a feature-rich, yet easy-to use configurable web interface that enables engagement with historical manuscripts, making collections visible, searchable, and increasing openness and visibility of materials. Across the 34 individual projects supported by read&search, Users can conduct comprehensive searches across large collections, using facets, fuzzy search, and probabilistic search over recognition lattices (Smart Search). This method indexes multiple word hypotheses per recognised line, enabling retrieval based on confidence-weighted alternatives rather than exact top-1 matches. Text can be overlaid on or viewed alongside page images for enhanced understanding. Additionally, this functionality is integrated with the Transkribus web-transcription interface for crowd-sourcing efforts. In 2023, read&search was upgraded to a new version of the product, Transkribus Sites ( https://www.transkribus.org/sites), with further customisation so that, for a yearly fee, Users can build their own websites and publish digital documents online, without the need for coding skills or institutional IT support, solving the problem of how to share work publicly without needing institutional infrastructure or technical support (although all content can be exported and hosted by Users themselves, if preferred). A total of 40 Sites are currently supported, including the Weisses Register ( https://app.transkribus.org/sites/weisses-register-zuerich), an index of the records of the Zurich State Archives from the Middle Ages to 1840, and the first twelve editions of *Küçük Mecmua* ( https://app.transkribus.org/sites/kucukmecmua.org/about), a seminal Ottoman Turkish weekly journal, essential for scholars of late Ottoman culture, literature and philosophy, published between 1922 and 1923. In this way, Transkribus Sites blends AI processes with the (traditionally more hands-on) building of digital scholarly editions, given it delivers:

an emerging type of digital edition: one that is produced directly from both the inputs and outputs of Handwritten Text Recognition, without the need for further processing, data transformation, or design and hosting. Taken together, they will eventually allow critical components to be added to this basic functionality. The development of these platforms is deserving of careful consideration regarding access, and opportunities, enabling a wider community to create digital editions with relative ease, especially as the functionalities of automated editions develop. ( Terras *et al.*, 2024).

Technical development of the Transkribus platform continues. In 2022, in response to user demand, Transkribus On-Prem was launched, to give organisations the ability to host the workflow on their own premises, in their own environment, where data never leaves their own servers and images are processed locally, for security purposes ( https://readcoop.eu/transkribus/on-prem). Research and development on improving ATR itself has focussed on a 5-based text transformer recognition model (a neural network that learns context and has superior performance on NLP and image processing tasks), which was dependent on READ-COOP Members sharing their ground-truth data - with consent and attribution - to improve recognition quality of the new models. The transformer technology takes more benefit from large datasets to enable both the training of large, general-purpose models, and higher recognition quality in specialised scenarios (Transkribus READ-COOP, 2023j). Transformer recognition HTR (TrHTR, adapted from TrOCR ( Li *et al.*, 2023)) ^27^ was launched in beta for testing in Transkribus in October 2022 ( READ-COOP, 2023g) followed by further optimisation including enhanced image pre-processing and improving performance for several languages. TrHTR was fully integrated into the Transkribus metagrapho API (which allows programmers to quickly integrate AI-based text recognition into existing workflows or tool chains, see https://readcoop.eu/api/) in 2023, with the release of the Text Titan recognition model ( READ-COOP, 2023h). A specialist Dutch-Language TrHTR model, Dutch Demeter I, trained on 18m words of data from READ-COOP Members including Stadsarchief Amsterdam and the Netherland’s Nationaal Archief, was released in February 2024, capable of an average CER of 4.9% ( READ-COOP, 2024d). Text Titan II, another TrHTR model, fine-tuned with over 400k Pages and 85M Words, and supporting 17 languages (German, English, French, Dutch, Italian, Latin, Spanish, Portuguese, Finnish, Swedish, Polish, Danish, Czech, Slovak, Estonian, Icelandic, and Hungarian) is currently in preparation ( Transkribus, 2024b). Access to transformer models is a premium feature, encouraging power-Users to take out a tailored subscription, with a range of new levels (Individual, Scholar, and Organisation) with different offers launched in 2023 ( READ-COOP, 2023i) and a Team subscription launched in 2024 ( READ-COOP, 2024c), reflecting user feedback requesting another tier. Similarly, development of field and table models for trainable analysis of complex layouts has taken place and is now integrated into the API and web app ( Transkribus, 2023d), with instance segmentation for table recognition planned for release. Alongside this, the creation of models by the community continues, including the launch in October 2024 of the first Arabic model for handwritten texts from the 17 ^th^ to 20 ^th^ centuries ( Transkribus, 2024d).

A range of technical developments and improvements are ongoing. The rebuilding of the back end of Transkribus, onto a new microservices architecture, began in 2022 and is a major ongoing project for the development team. Trainable Named-Entity Recognition is under development, as is a commenting function for collaborating on documents, currently in Beta testing as of October 2024 ( READ-COOP, 2024b). There are also plans to support and grow the community, fostering connections between Members with a new Transkribus Connect platform, and the launch of a more advanced investment vehicle with premium memberships for large institutions. Continual development and improvement such as these are essential for the software-as-a-service offer, to keep Transkribus in a market-leading position, given the growing demand for ATR in historical document digitisation but also in healthcare, banking and finance, the legal sector, and education. Development is essential to retain Transkribus community support: this also benefits READ-COOP in understanding community needs, gathering input on future plans and Transkribus’ technical offer, rapidly communicating any platform issues, and providing a mechanism for democratic decision making.

### 5.7 Impact

***5.7.1 Scholarly impact.*** The first-mover advantage of the Transkribus platform ( Kerin *et al.*, 1992; Papachristos & Suarez, 2022), combined with effective retention mechanisms, have resulted in a combined network effect ( Belleflamme & Peitz, 2018) that has seen widespread uptake of the tools provided by Transkribus, which are therefore having an increasing amount of impact on the historical, academic, and Gallery, Library, Archive, and Museum (GLAM) sector. By the end of 2020, Transkribus and its tools were credited in 381 academic publications, including journal articles (163), conference papers (80), and policy documents (31) ( Nockels *et al.*, 2022). Celebrating this, in 2020, Transkribus won the Horizon Impact Award, the European Commission’s initiative to recognise and celebrate outstanding projects that have used their results to provide value for society ( EU Science and Innovation, 2020; European Commission, 2022). Since then, Transkribus has supported thousands of further projects undertaken by a range of individuals including students, scholars, and hobbyists worldwide, in hundreds of institutions and private collections, covering a huge range of scripts, languages, and dialects. For example, Transkribus has been used for: accurate transcription of bilingual Evenki-Russian manuscripts including folklore, personal narratives, and shamanistic rites ( Arkhipov *et al.*, 2021); to develop a ground truth dataset for Sanskrit and Newar Nepalese manuscripts in Pracalit script, supporting others working in Indology and Newar studies ( O’Neill & Hill, 2022); transcribing Old Nubian and multi-script text from Medieval Nubia to enhance accuracy of mixed-text transcription in Africa ( Miyagawa, 2023); and to support the analysis of baptism records in Portuguese, identifying slave owners and slave families in 18 ^th^ century Brazil ( Nascimento, 2024).

**Figure 11.**
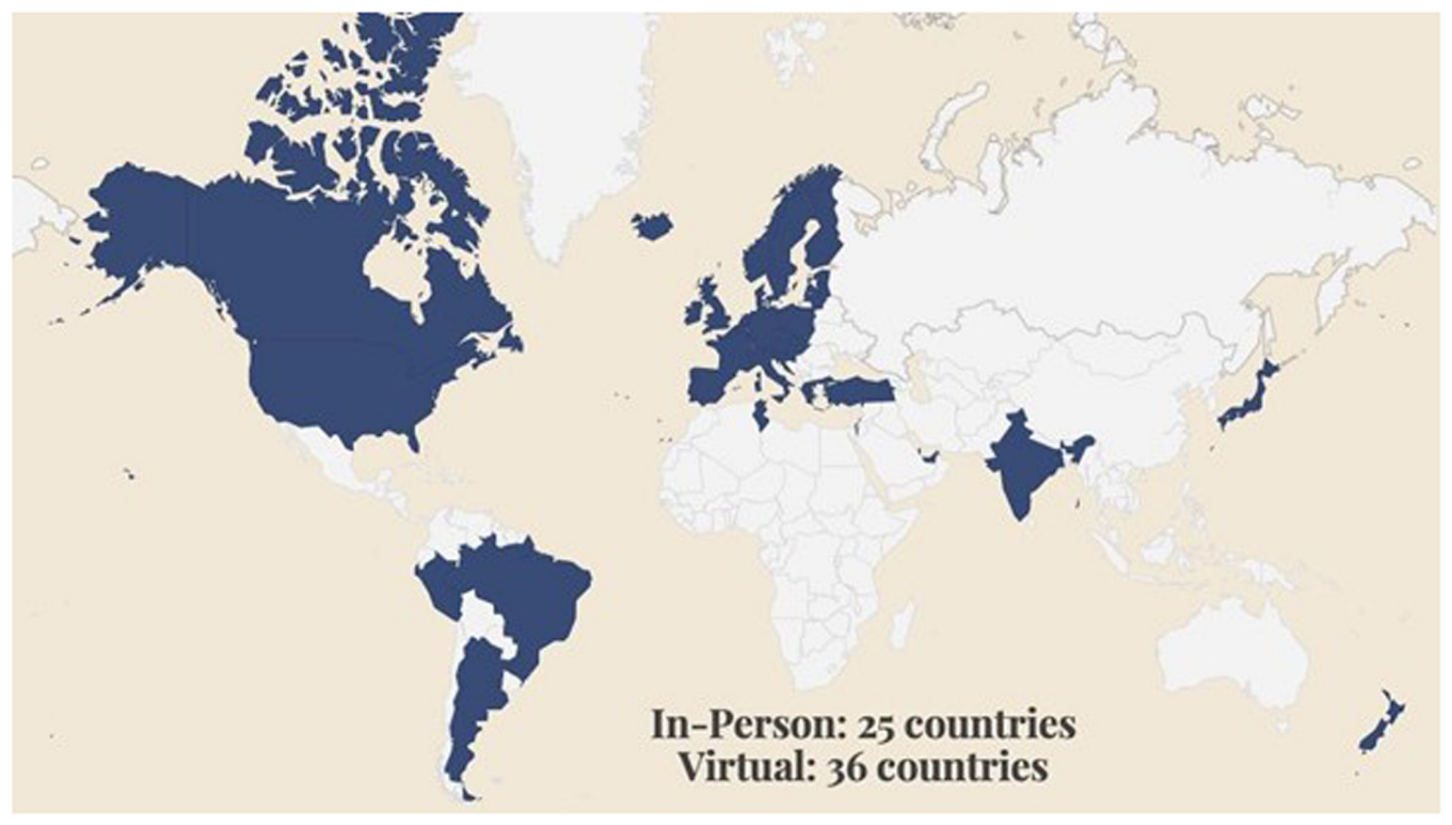
Home countries of attendees (in person and online) Transkribus User Conference 2024.

In many ways the overall user community is now too large to document, but the cooperative structure supports READ-COOP to meaningfully engage with as many core and engaged Users are possible: at the 2024 Transkribus User Conference on the 15 ^th^ and 16 ^th^ February, University of Innsbruck, 80 speakers from 40 countries presented, with in person delegates from 25 countries, and online access from 36 countries ( READ-COOP, 2024a) (see Figure 11). Although many software companies hold annual user conferences, the cooperative framework provided by READ-COOP allows these delegates to become integral contributors to the infrastructure, year-round, while using the TUC in person meet ups to strengthen relationships.

***5.7.2 ATR data sharing.*** A further impact of the collaborative approach to ATR is the publishing of licensed “ground truth” ^28^ ATR data for Transkribus community reuse. As of October 2024, READ-COOP hosts 230 currently available, licensed, internally available AI models for use only with READ-COOP infrastructure, so that others can “benefit from the network effect and save work and time” ( READ-COOP, 2023c), thus making it easier for others to undertake transcription of their digitised content with Transkribus. Recent releases include PyLaia models The Dutchess I., “a comprehensive solution for deciphering historical documents from the Low Countries” ( READ-COOP, 2023d), and The German Giant I., “A combination of models containing German language Kurrent and Latin script” ( READ-COOP, 2023e). The availability of such models to registered Transkribus Users highlights the significance of shared community infrastructure, but also demonstrates where criticism of the closed nature of the platform comes into play, given these models cannot be exported from READ-COOP infrastructure, and the weights, code and documentation of the model are not published publicly (which would be a prerequisite for an open model in AI discourse, see Widder *et al.*, 2024).

However, Transkribus collection owners can export their training text recognition and segmentation model data to the wider HTR community, primarily through HTR-United ( https://htr-united.github.io, Chagué & Clérice, 2023), established in 2020 to share ATR datasets. As of October 2024, 22 out of 102 datasets on HTR-United were created with Transkribus. However, the lack of best practices or infrastructure to recognize data annotators’ contributions remains a growing ethical concern within itself. The READ-COOP community plays a central role in Transkribus’ success, yet the visibility of community contributions to AI models remains an open discussion ( Romein *et al*., 2024). There is a tension between creating AI tools and being transparent about data annotator contributions. Since its inception, Transkribus has adopted a zero-knowledge model for training to maintain ethical consistency and fairness, avoid reinforcing academic hierarchies, ensure legal clarity under GDPR, and uphold product integrity, ensuring training inputs remain untraceable to specific documents. We openly inform our Users that private data may contribute to models benefiting others, and any Member has extensive rights to influence the technology's development and commercialization approach. However, contributors are listed for models with active collaboration and consent, such as Dutch Demeter.

Discussions on the sharing of Ground Truth data have resulted in guidelines for appropriately citing ATR data, models, and volunteer contributions, developed by Transkribus Users after a TUC 2022 workshop. These guidelines address “broader issues surrounding the use of Machine Learning in archival and library contexts, and how the community should begin to acknowledge and record both contributions and data provenance” ( Romein *et al.*, 2024, 1). We advocate for systematic anonymization with optional opt-in attribution as a responsible long-term framework and are exploring ways to implement this more meaningfully.

***5.7.3 AI and the GLAM Sector.*** The impact of Transkribus may go beyond the application of the platform itself: this has been noted by many writing on the roll out of AI in the GLAM sector. Automated Text Recognition (ATR) technology “is now a mature machine learning tool, becoming integrated in the digitisation processes of libraries and archives, speeding up the transcription of primary sources and facilitating full text searching and analysis of historic texts at scale” ( Nockels *et al.*, 2022). Transkribus is therefore an example of how AI can be integrated into cultural heritage organisations, in a way that is acceptable for both institutions and users: “In the case of Transkribus, end-users are empowered” ( Lee, 2023). It therefore gives a concrete instance of how GLAM can make the most of AI and machine learning in a responsible and ethical way (while in a time and media context of “AI Panic”, see Leaver & Srdarov, 2023). Transkribus demonstrates that cultural heritage institutions must “Develop workflows in partnership with disciplinary researchers that can identify, extract, and make machine-actionable data from general and special collections to fuel library experimentation and research activity on campus” ( Padilla, 2019, 16). These type of established workflows “anchored in core activity will begin to show the potential of algorithmic methods to assist with processing collections at scale; alleviate concerns about sustainability by proving impact on core operations; and help smooth the path to integrating probabilistic data in a discovery system” ( Padilla, 2019, 15). The fact that Transkribus is a “gold standard” ( Ryan, 2023) and a “prime example” of the type of AI that “is being increasingly embedded in academic libraries tools and services” ( McAllister *et al.*, 2022, 247) paves the way for others, as does the methods it has adopted in engaging with its community:

In addition to its outreach to scholars, the Transkribus project describes itself as a resource for libraries seeking to mobilize a local community of users (librarians, scholars, students) to rapidly develop an HTR model for a local collection and thereby enrich its data, as well as for computer scientists who need an environment to test tools in development. As such we might look to this project as a model for motivated crowdsourced engagement… in order to build domain data.”… There is a “need among scholars and libraries for more domain-specific training data, if we hope to see a wider investment in ML projects. Transkribus provides a compelling model for community-driven efforts to create, gather, and share training data, as well as the potential for crowdsourcing for rapid dataset development. While Transkribus focuses on handwritten text recognition—alongside some OCR—its platform and community-engagement models could be adapted across a wide range of domains and ML projects ( Cordell, 2020, 25–6).

Upon the 5 ^th^ anniversary of READ-COOP in July 2024, Dr Günter Mühlberger, the Chair and Founder, said:

After 5 years of READCOOP I am very proud that “our child” is growing and that Transkribus is now a kind of “standard” in the community. The amount of people and projects who are actually using the platform is enormous and exceeds by far expectations. Also that 200 Members have joined the coop is far beyond what I had envisaged.
There is of course more to be done. E.g. I am dreaming of a distributed structure for storing and processing documents and data in a decentralized way by using also infrastructure from our Members. On the downside I am bit sad that we were not able to continue working with some of the original partners in the READ project, some are now even competing with us ^29^. Anyway, I believe that we are really making a fair offer, and welcome engagement with a range of providers and platforms. The new business and pricing model is a big progress to what we had before, so that I am very optimistic that we are able to expand our services, that our Users and Members are loyal to Transkribus and that they appreciate especially the personal support we are providing ( Muehlberger, 2024).

However, although Transkribus and its results are now becoming embedded into the scholarly environment, there has been no in-depth published commentary by other researchers so far about the fact that Transkribus is run by a cooperative. This case study has shown that mechanism is fundamental to its relationship with community, and to the provision of its infrastructure. To further show evidence of engagement with the user community, it is useful to consider the opinions of Members of READ-COOP themselves.

## 6. Surveying READ-COOP membership

Opinions of Members on the functioning of READ-COOP, and wider opportunities for cooperative AI infrastructures, were gathered via an online survey between 21 ^st^ September and 21 ^st^ October 2023, revealing various themes and emergent issues. No paper authors responded to the survey to ensure impartiality. Quoted text is given a number corresponding to the order in which READ-COOP Member (RCM) submitted their anonymous response (although due to some traceable, identifiable comments we have not released the survey data for GDPR reasons). It should be stressed that the survey gained responses from a smaller sample of self-selecting founding Members, with the results foregrounding their experience. A future study will survey READ-COOP Users, and future work could also interview private Members of READ-COOP to establish their relationship to the platform, and how that intersects with the type of institutional support they have access to.

### 6.1 Response to survey

The survey received 34 responses, comprising of 15 individual (private) Members of READ-COOP, 13 representatives of Members organisation, and 6 Members who are both private Members and organisational representatives (out of a possible total of 73 private Members and 90 institutional memberships at the time of data collection). In total, 29% (n=21) of private Members responded to the survey, and 21% (n=19) of organisational representatives responded. Research indicates that a 20–25% response rate in smaller community sizes can provide confident estimates ( Fosnacht *et al.*, 2017), however, qualitative analysis of the comments is essential given the smaller sample size, and it should also be noted that any comments are contextually linked to the operations of the cultural and heritage sector.

Of those responding, 35% had been Members since 2019 (n=12), 21% (n=7) since 2020, 12% (n=4) since 2021, 12% (n=4) since 2020, and 21% (n=7) since 2023, suggesting that longstanding Members of READ-COOP were more likely to self-select to reply. Over half of the respondents became READ-COOP Members within the first 18 months of operation.

### 6.2 Involvement in READ-COOP activities

Within our sample of self-selecting Members who responded to the survey, 94% (n=32) were aware that READ-COOP is set up under a cooperative legal framework (the remaining 2 Members were unsure). 53% (n=18) of Members felt very informed about the role of the cooperative, and how it relates to Transkribus. 41% (n=14) felt a little informed, and the remaining 2 Members were unsure. Only 2 Members (6%) felt they did not get enough information about how READ-COOP operates, although a further 7 (21%) were unsure. 27% (n=9) reported that they were provided with lots of information, and 46% (n=15) said they had sufficient information. 84% (n=27) of survey respondents had attended at least one monthly online READ-COOP Members Meeting, and 69% (n=22) had attended the online annual General Meeting (see Table 3 and its discussion which demonstrates that on average, approximately 25% of Members attend monthly meetings and 42% attend AGMs: this indicates our sample is a set of very committed Members). 81% (n=26) discussed READ-COOP activities in the Members Slack channel. 56.3% (n=18) had attended the Transkribus User Conference in person, with 19% (n=6) attending online. 59% (n=19) had used Transkribus tutorials or other online training, and 28% (n=9) used the Transkribus discussion groups on Facebook. An informal WhatsApp group was also reported as a means of communication (6%, n=2).

### 6.3 Reasons for becoming a READ-COOP member

A thematic Content Analysis of free-text descriptions of why Members chose to join READ-COOP reveals nine core reasons for membership (see Table 5).

**Table 5.** Thematic analysis of reasons given for joining READ-COOP. Members could mention more than one.

Reason Given for Joining READ-COOP as a Member	Number of Responses	Percentage
To save money via members’ discount	7	21%
To support the further development of READ-COOP services	7	21%
To show support/belief in READ-COOP	7	21%
For good purpose and to participate in the community	6	18%
Improved sustainability of infrastructure via cooperation	3	9%
Better opportunities for collaboration	3	9%
Ability to directly influence future direction of READ- COOP	3	9%
To have research support	2	6%
Access to cutting edge AI	2	6%

While saving money is a key reason for membership, it was also notable that respondents were heavily invested in community participation and supporting further development of the infrastructure. Membership discount is amongst the top reasons for engaging in READ-COOP “Provide access to discounts and services otherwise unaffordable (or less affordable) to individual researchers at our institution” (RCM29); “For the discount on Transkribus invoices as well as to support and influence the goals of the cooperation” (RCM 16). However, equally important weighting is given to showing support for READ-COOP: “supporting a great initiative” (RCM20); and to support the further development of its services “being part of an ongoing process to improve AI text recognition and unlock old documents” (RCM12); “I want to help advance the use of HTR in genealogical research” (RCM19). Community and participation was a strong draw “wanting to participate actively in making things better” (RCM5); “Best possible human component in the Coop” (RCM7), with a view that READ-COOP provided opportunities for networking and collaboration “In essence, joining READ-COOP allows institutions to tap into a network of knowledge” (RCM 30) which also leads to sustainability: “I use Transkribus a lot and did not want the technology to go to waste” (RCM15); “To support this initiative stays free of corporate or other influences” (RCM21). A few Members wanted to influence future developments “I’m using it for my research and I contribute a lot to the project, so I want to have a say” (RCM33), or to have access to support and cutting edge AI “early access to new features” (RCM29). There is a prominence of participatory and contributary activity throughout all of these comments, stressing the engaged nature of the community.

### 6.4 Benefits to READ-COOP members

Private Members were asked if they had received any personal benefits from being part of READ-COOP. 61% (n=22) identified benefits, with the most popular being Networking (55%, n=13) “it is a community and I really appreciate being with people who are working towards the same goals” (RCM22), “I’ve met a lot of great people that are highly interested in text recognition/working with cultural heritage” (RCM27), “I found a family who shares a common vision with impeccable respect to each other” (RCM30); then Technical Support (33%, n=8), “direct troubleshooting and bug fixing” (RCM33). Others mentioned Learning (17%, n=4), “am learning far more than I would had I not joined” (RCM11). Each of these themes was mentioned by one Member: Discounts, Reputational Benefits, Access to events, Project Planning support, and Positive Feedback “It has been a great benefit to witness READ COOP stays independent” (RCM21). Four Members (12%) said they saw no personal benefits “No - did not look for it either” (RCM6): indicating that the cooperative model distances the user from a transactional relationship, being happy to contribute despite not perceiving personal benefit.

Members were asked what benefits there were for an institution becoming part of READ-COOP. 89% (n=30) mentioned discounted credits, but also popular were the sharing of best practice (76%, n=26), being part of a network (70%, n=24), and getting access to information and support (67%, n=23). Comments included “the institution mostly joined because of the discount but there was also the kudos in being an early founding organisation” (RCM18), “being part of a community, sharing experiences and getting help in projects by other institutes who struggle with similar problems. Working as a group of specialists on one project. Getting credit discounts” (RCM14), and “I think the big one is the ability to steer the development of the tool, for instance certain parameters or features, as part of regular member discussions. Having early access to new developments and being kept in the loop at the TUC especially” (RCM3). One Member commented “There is a lot of concern about ethical AI and so my organization is more comfortable with supporting a cooperative structure” (RCM32).

Members were asked if there were any downsides to being part of the cooperative. 24% (n=8) said no, with a further 12% (n=4) saying they could not think of any. However, 24% (n=8) mentioned that costs were an issue: “The usage for academic large-scale projects is usually too expensive if experimental parts for training/several rounds of recognition etc. are foreseen” (RCM17), “Transkribus is expensive to use, and you don’t own the model data” (RCM25), “Having to explain the contribution fee frequently to my institute/ financial department” (RCM13). Concerns about costs to access the infrastructure have been a recurrent theme since Transkribus started to discuss moving from a free-to-use tool to one based on a credit system ( Nockels, 2024; Seaward, 2019; Terras, 2022). One Member commented “I wonder how sustainable it is - it is dependent more on institutions becoming part of the coop. There is the issue of institutional inertia and the lack of uptake. Any keen staff member(s) has/have to persuade an organisation to join. This is too hard without support. Some organisations understand and can see the strategic benefits of Transkribus, but most don’t. READ-COOP could do more to provide resources and support for getting buy-in- for individual staff members, and also for organisations... the Coop could do more” (RCM22).

### 6.5 Understanding READ-COOP operations

Members were asked if they knew of how READ-COOP planned to reinvest profits into its infrastructure. 26% (n=9) said they did not know “No but I am happy they exist and that this concept lives on” (RCM21). Of the 59% (n=20) who did know, many listed specific technical features development including “Improving layout/structure recognition, integrate new recognition engines, integrate large language models, Handwritten Music recognition” (RCM33), with one commenting “it is described and outlined during the monthly member meetings” (RCM34). Further information requested from Members included understanding more about how strategy is developed, learning where READ-COOP records are kept and how to access them, “more transparent communication when something is not working” (RCM28), and being kept informed about company priorities: “I would like to know how many people work in sales and advertising compared to operating the infrastructure and development of front-/backend” (RCM27) and “I would like READ-COOP to be more transparent in communicating what kind of user needs they prioritize the highest. E.g. does the cooperative see itself as mostly focused on the needs of larger institutions, such as national archives, or does it mostly seek to fulfil the needs of smaller groups, such as museums and private users?” (RCM25). Throughout the survey there were comments on the lack of open publishing of the code and datasets that underpin Transkribus (it is not an open-source tool, given its datasets contain the unique IP that the cooperative depends on to trade): “The shared access is only within the Transkribus universe and therefore not really shared access or machine-learning in a bigger picture. I understand why it’s not fully open. Maybe you could communicate those reasons more?” (RCM5). It is here that there is a tension between the Cooperation and Concern for Community cooperative principles: indicating the limits there are for open access and open science within AI development ( Bostrom, 2018). There is a very real threat from dominant extractive AI providers who could ingest any openly published code or data and offer similar tools to extend their monopoly and quash competition: AI cooperatives must guard their intellectual property whilst also attempting to be ethical and inclusive, and this tension is felt by READ-COOP Members.

### 6.6 Relationship to READ-COOP

**Figure 12.**
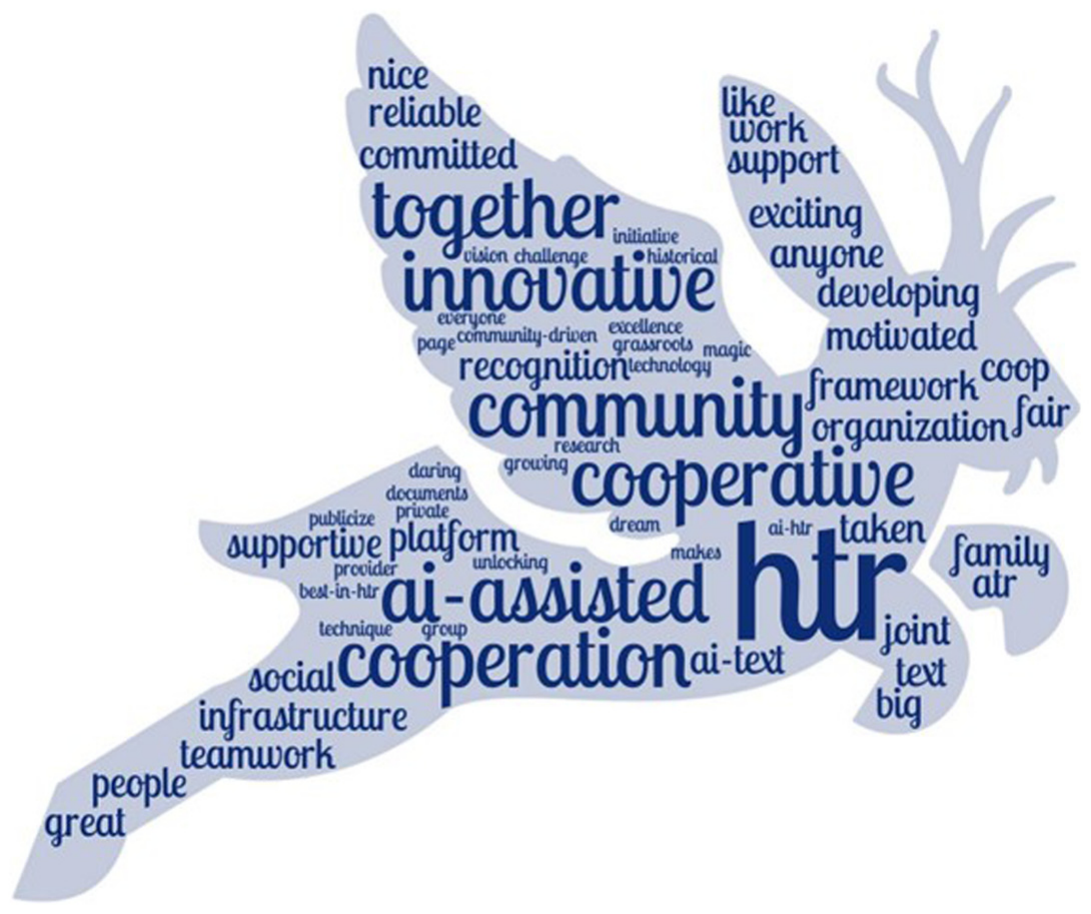
Word cloud generated from Member’s descriptions of READ-COOP, in the shape of Wolpi, Transkribus mascot.

Members were asked to describe READ-COOP in three words, with 28 Members providing responses. The resulting word list was used to generate a word cloud visualisation which stresses the committed community, and motivations for supporting the AI ATR infrastructure (see Figure 12).

These Member-provided words align closely with the seven cooperative principles, when all words are thematically clustered (see Table 6). The emphasis on inclusivity, teamwork, economic participation, autonomy, and social responsibility demonstrate the importance of the cooperative approach to READ-COOP Members. Words which do not align with the cooperative principles cover both the AI technologies, and the excitement that accompanies being part of the initiative.

**Table 6.** Clustering of Descriptive Words provided by READ-COOP Members, aligned to Cooperative Principles ( ICA COOP, 2024).

Cooperative Principles	Aligned Words From Member 3 Word Comments
Voluntary and Open Membership	Community (x5), Community-driven, Everyone, Fair, Open, People
Democratic Member Control	Cooperative (x2), Cooperation, Framework, Teamwork, Vision
Member Economic Participation	Challenge, Committed, Group, Initiative, Joint, Share
Autonomy and Independence	Independent, Autonomy, Private, Self-help
Education, Training, and Information	Developing, Education, Innovative (x2), Training, Learning, Research, Technique, Technology
Cooperation among Cooperatives	Network, Organize, Organization, Partnership, Publicize, Work
Concern for Community	Family, Grassroots, Social, Support, Supportive, Together (x2)
Other	AI, AI-assisted (x2), AI-HTR, AI-text, ATR, Best-in-HTR, Big, Daring, Documents, Dream, Exciting, Excellence, Great, Growing, Historical, HTR (x4), Infrastructure, Like, Magic, Motivated, Nice, Platform, Provider, Recognition, Reliable, Taken, Text, Unlocking, Up

When asked how being a Member of READ-COOP made them feel, 94% (n=32) said “engaged”, 68% (n=23) said “included” and “supported”, 38% (n=13) said “valued” and 32% (n=11) said “trusted”. From the list of more negative options, 9% (n=3) said they felt “annoyed” and “ignored” and 1 person (3%) each said READ-COOP left them feeling “confused”, “isolated” and “overwhelmed”. Overall, then, READ-COOP membership would appear to be a positive experience for Members.

Members were asked if a cooperative structure made technology providers more transparent (62% said yes, n=21), trustworthy (59% said yes, n=20), approachable (53% said yes, n=18), responsible (50% said yes, n=17), sustainable (50% said yes, n=17), ethical (47% said yes, n=16), efficient (26% said yes, n=9), effective (21% said yes, n=7). Only 9% of respondents (n=3) said “none of the above”, suggesting that there are elements of cooperative organisation practices which benefit the relationship between users and technology providers, although some do not engage. It is interesting here that Members do not choose “efficient” and “effective”: suggesting that they do not see the cooperative as benefitting the product, so much as the relationship between them and the platform. When asked if this relationship felt different to that of other technology providers (who are not governed by a cooperative), 79% (n=27) said there was significant differences: “Yes. I am part of it and I have a say on its program” (RCM33), “Yes, greater level of information than other freemium/for-profit providers” (RCM8), “I think the technology shines more and therefore develops faster and more robustly in this environment” (RCM18), “In the case of our text recognition, other for-profit providers provide us with good troubleshooting and support (through direct lines of contact), this is the same case with READ-COOP. The fact that Transkribus appears more malleable as a tool and is constantly being innovative makes it feel like users have an increased say” (RCM3). There was only one negative response: “We have the General Meeting, among others, which we would not have with for-profit providers, but sometimes the meetings seem also a bit like a marketing event. So there are also similar topics than with for-profit providers” (RCM28).

Members were asked if the cooperative framework behind READ-COOP gave them comfort regarding the long-term sustainability of Transkribus. 47% (n=16) said “Yes – a little”, and 26% (n=9) responded “Yes – a lot”. However, 9% (n=3) thought that this was no different to any other technology provider, and a further 9% thought that the cooperative framework would have no effect on Transkribus’ sustainability.

### 6.7 READ-COOP Member views on cooperatives and AI infrastructures

When asked if cooperative business models would benefit other AI providers, READ-COOP Members responded 44% (n=15) “yes”, 29% (n=10) “maybe”, with 24% (n=8) “unsure”: there were no respondents who answered “no”. Their views on why there are not any other cooperative technology providers, and in particular no other Machine Learning/AI cooperative infrastructures, were many and varied. Profits were most commonly raised (15%, n=5) “Because most technologies are products and services created by large companies. Coops are a different model. I am not sure with machine learning/AI it is the most profitable given that large companies are seeking profits for shareholders” (RCM22), “People are extremely interested in exploiting the commercial potential of technology they help develop” (RCM11). Others pointed out the high initial costs for AI/ML R&D, “High Initial Costs and Complexity of Development” (RCM7) and lack of access to, and associated costs of, large-scale compute. Individual responses included the fact that many start-up initiatives are “bought by commercial empires or disappear at an earlier stage” (RCM33), suggesting that there is a hope that the cooperative model could provide longevity and stability. A range of responses suggested there is a different focus of cooperatives “companies and their investors focus on high earnings where as in a coop the focus is on the best value” (RCM24), and that cooperatives were not well-known “in a society where the personal success is most important a cooperative model is not so attractive and certainly it is not promoted in the public (and universities, funding agencies, etc.)” (RCM4). Respondees were keen to situate the work of AI cooperatives alongside major technology providers “Another reason could be that cooperative technology is sort of in between two worlds. I think there are currently two major positions that AI companies take in releasing technology: proprietary technology and open technology. With closed technology or AI models there are benefits in the form of controlled vision and financial security. With open technology or models the benefit is in letting a community freely use and quickly improve it. Cooperative technology could be somewhere in between: too open to benefit from centralized control and too closed to benefit from true open community development” (RCM16).

A third of respondents, (35%, n=12) believed that the development of other AI cooperatives would allow for greater user engagement, inclusion, and co-creation: “I believe so, great interoperability between tools would perhaps emerge also. The notion that we are paying for a service and expect a certain response is quite limiting, without having a direct say in the operation of a cooperative” (RCM3). However, just as many respondents did not know if this would be possible “many users don’t have time to get really engaged in a product; they just want to use the product due time limits in delivering a result. Sadly that is the reality in the business world” (RCM14). There was hope in exploring this approach, though: “I hope that READ-COOP can provide a framework for successful cooperative AI structure, and that other tool providers take note” (RCM3) but also a note of caution: “how can we change this now, is it too late?” (RCM18).

### 6.8 Survey summary

Given the relatively low number of respondents to the survey, mostly from early adopters of the platform, these are qualitative responses, however the comments made by READ-COOP Members show a nuanced understanding of its cooperative structure. Members expressed a high level of engagement and a sense of inclusion within the cooperative’s activities, which reflects positively on the organizational structure. Notably, respondents saw the cooperative nature as fostering transparency, trustworthiness, and approachability, while aligning with their needs and values. For READ-COOP Members, enhanced user involvement in decision-making processes strengthens the commitment to developing AI technologies that address real community needs while also ensuring broader participation in governance. Despite challenges regarding cost, and questions regarding openly publishing datasets, the majority of Members who responded to the survey recognize significant benefits, including discounted access to the infrastructure, networking, technical support, and influence over the platform’s direction, showcasing a responsible, strong community-driven infrastructure that could serve as a model for future AI initiatives, particularly given these activities map so strongly onto Floridi and Cowls’, 2019 principles for responsible AI (see Table 7), while additionally stressing the benefits of Member Economic Participation and Concern for Community which are embedded elements of the Cooperative Principles ( ICA, 2024).

**Table 7.** READ COOP activity mapped to Floridi and Cowls (2019) 5 principles of AI in society.

AI In Society Principle	READ-COOP Activity	READ-COOP Member Comments
**Beneficence**	Promotes cultural understanding, preserving heritage, and enhances historical research.	Members appreciate democratization and accessibility: “being part of an ongoing process to improve AI text recognition and unlock old documents” (RCM12).
**Non-Maleficence**	Establishes security and data integrity through democratic decision-making.	Members value the security and integrity provided by the cooperative control: “To support this initiative stays free of corporate or other influences” (RCM21).
**Autonomy**	Allows direct input into AI development and governance, influencing operational strategies.	Members are involved in shaping the tools and services that affect their work and community, reflecting empowerment and inclusiveness: “I am part of it and I have a say on its program” (RCM33).
**Justice**	Ensures equitable access to technology, fostering diverse cultural contributions which facilitate equal benefits from AI advancements.	Members appreciate inclusivity and fairness: "To make research and cultural insights through AI more accessible" (RCM25).
**Explicability**	Promotes transparency in operational processes and through Member meetings and open communications.	Members value transparency and stating a desire for clear and open AI practices "Greater level of information than other freemium/for-profit providers" (RCM8).

It is worth comparing this level of engagement and responsiveness to the Member community with that on offer from big AI, where notions of user input are barely visible, and often amount to offering individuals the opportunity for retrospective feedback, if they can locate the mechanism to do so ^30^. We argue that the cooperative approach gives core users the option to engage with the running of the infrastructure they depend on, and this opportunity for meaningful engagement is a core contribution to more transparent and responsible AI business practices. However, while many Members of READ-COOP stress the benefits of being part of the cooperative structure beyond merely accessing discounts when using the platform, there are also a significant percentage that do not meaningfully engage with cooperative activities, and some who remain sceptical that these activities are any more meaningful than standard software marketing approaches. We are also aware that our responses are entirely context-specific, working within the cultural and heritage sector, and so this may not translate elsewhere, and we also note that future work necessitates us asking these questions of our Users, as well as our Members.

## 7. Discussion

ATR and machine learning are very particular sub-strands of the wider Artificial Intelligence field. READ-COOP represents a relatively small community worldwide committed to a highly focussed, defined task: the ATR of historical documents, particularly for the library, archive, and museum sector, and researchers which depends on accessing their content. READ-COOP is dependent on the investment and support of many organisations: secure and sustained “memory institutions” whose role it is to steward the past for the long-term. There is something unique about building an agile, cooperative technological tool serving across this long-standing domain, rooted in a community with a history of civic and democratic activism ( Kranich, 2020), and engagement with digital scholarship ( Mackenzie & Martin, 2016). The cooperative structure may be as much of a support for community-embedded digital innovation, as well as ethical governance: particularly when there is a clear sense of purpose to deliver a high-quality tool responsive to user needs. However, the cooperative model does put all resources into one basket: the co-ownership structure means that it would be impossible to pivot to a conventional business model or be bought-out by another business in the case of financial issues, given every single co-owner would have to agree to this. However, READ-COOP is unusual as a cooperative: benefiting as it did from a level of initial grant-funding and support from university infrastructure that is beyond the reach of most platform cooperatives: not only is it unique, it is an outlier, and considering this may have ramifications for others wishing to adopt cooperative approaches to AI, or funders wishing to support innovation from focussed research projects.

The size of Transkribus’ user base, and the income generated to keep the infrastructure operational, means READ-COOP is an AI minnow to Apple, Microsoft, Alphabet, Meta and OpenAI's pikes. Transkribus has a fraction of big AI’s resource or market share, and a completely different vision. Unlike leading generative AI providers, Transkribus and READ-COOP aim to integrate the Member-base into decision making and re-invest profits into shared goals. However, the large-scale compute, datasets required and created, and user-facing platform developed by Transkribus has much in common with other current commercial AI systems (and a comparison with other smaller scale HTR providers would be informative). The success of Transkribus demonstrates that specific and targeted AI and ML tools can have closer links to a target community, and a more ethical technological approach, than larger, general tools. Transkribus is therefore part of much wider and stronger tides in the modern technical and socio-political world, questioning how to build, maintain and grow ethical digital infrastructures in responsible and sustainable ways. Grappling with the underlying business models of AI providers is essential, and we must start to question our growing dependency on easy-to-reach-for platforms provided by technological monopolies, while also providing mechanisms to engage specific communities in successful tool delivery.

The act of founding READ-COOP as a cooperative to deliver Transkribus in its post-funded phase has been both an innovative and hopeful act, demonstrating the possibilities for using cooperative methods of governance in platform AI: “To build a co-op is to challenge old ways of thinking about “property” and “development” and contribute to new narratives about community rights, sustainable development, and the satisfaction of basic human needs” ( Scholz, 2023, 178). This challenges the domination of corporations “that are destroying their own lives, the lives of others, and even the material conditions of their existence in order to satisfy their craving for profits” ( Davies, 2024, 267). When it comes to AI, “we need to focus on the questions of use, misuse, and analysis of data, and the social systems around that” and question “those who build the systems that our future societies will run on” ( Milne, 2020, 231) to avoid “systematically building a tougher system for future generations to contend with” (ibid, 247). As will all corporate activities, AI needs “diversity rather than uniformity in corporate governance, and we need it in all its various manifestations – ownership, board structure, and incentives – to be focussed on promoting the full breadth of corporate purposes and not the single goal of shareholder value. It is the basis on which the purpose and values are instilled throughout the organization and without it management cannot deliver on purpose” ( Mayer, 2018 115). Calling for greater democracy in every area of life will help “wrest spaces back from the market into genuine public ownership” ( Segal, 2023, 179): “if we are to avoid a calamitous future, we have to move away from a world based on private accumulation, and replace it with a politics of care, solidarity and equality” (ibid, 186). Framed in these terms, READ-COOP indicates an alternative future for AI platform governance and sustainability, and one that provides the mechanism for defined, or even marginalised groups, to determine the direction of the technologies they depend on ( Birhane *et al.*, 2022), while supporting technological innovation that responds to a particular sector’s needs.

However, while READ-COOP is now one of 44.1% of companies that survive their “difficult” first five years ( Glover, 2019), this does not mean it will survive into perpetuity. Rapid developments in the AI space, particularly with the developments of LLMs having success in handwriting recognition ( Ravey, 2024), are challenging Transkribus’ status as the major user-facing provider of ATR (although current provision does not yet allow the same quality or quantity of HTR for handwritten historical documents as Transkribus, nor support for community). READ-COOP’s cooperative status means that, should the company ever face extensive liquidity issues, there is not an easy option to sell to an investor, so there is no financial safety net beyond asking Members for further share purchases and membership options. (This means it also cannot do an “OpenAI” and pivot into returning profits to major investors). However, the community that surrounds, utilises, and financially supports Transkribus has a vested interest in seeing it succeed, and there is no doubt that the cooperative model adopted has helped the survival, development, and growth of the ATR infrastructure thus far.

Cooperatives that host AI and ML infrastructures remain rare: for highly motivated individuals the setting up of a cooperative may not be as attractive as the establishment of a private or limited liability company, due to the current possibility of market ownership and untold potential future riches, prioritising those with the shareholder profit motive. However, for public institutions (such as universities), a cooperative model will likely be more attractive if the benefits were more widely understood ( Thorpe, 2019). There is no doubt that the current R&D and set up costs for technology platforms are prohibitively large, and that start-up costs for developing public-facing AI platforms (such as general model creation) are restrictive. Alongside this, there are pressures on universities to engage in “entrepreneurial discovery and exploration of market opportunities” ( Compagnucci & Spigarelli, 2020), and much commercialised research is originally linked to grant-funded activity ( Lockett *et al.*, 2003). READ-COOP’s starting point was inheriting a successful technology that had been built during 6 years of EU funded research (totalling €10.6m public investment) supported by university infrastructure, that could then be commercialised (which, as we noted above, makes it an outlier, although this could be reproducible in principle via grant funding or crowd-investing). However, we argue that research funders should prioritise cooperative business models when supporting entrepreneurs to establishing companies from publicly funded research projects in order to support specific tools that answer the needs of specific user communities. This could ensure more transparent, responsible, and sustainable business practices, but requires a redefinition of what it means to set up a successful business in a post-funded phase. Research funders may have to mandate that entrepreneurs in receipt of public funding must consider alternative models of ownership including shared and co-ownership. In the case of AI, this should also add to existing ethically aligned design strategies ( Gardner *et al.*, 2022), further encouraging the building of trustworthy and responsible systems ( Université de Montréal, 2018) and moving beyond the capitalist AI-hype bubble ( Naughton, 2024), to provide much-needed sustainable ML infrastructure for definable beneficiaries.

Establishing a sea-change in corporate AI governance towards cooperative principles will, in itself, require support and encouragement. Various cooperative programmes currently exist, such as the UK Cooperative Bank’s UnFound programme, which provides business support to platform coop founders ( https://www.uk.coop/start-new-co-op/support/start-platform-co-op), and the US based Start.Coop, which provides support, training, and networking to strengthen the cooperative ecosytem ( https://www.start.coop/about). However, we argue that universities, research funders, technology incubators, and policy-makers have not yet understood the benefits that cooperatives can provide, the appropriateness of the business model particularly for supporting collaborative and responsible approaches to AI and ML, and the need for support, education, and resourcing of this space. We call upon these bodies to start actively supporting and promoting this alternative method to fund and maintain technology, and to provide resources that prioritise shared prosperity, particularly when these tools respond to needs of particular, specific sectors and their communities. The establishment of platform, AI, and ML cooperatives that emanate from research communities should be seen as much of a success as venture-capital built start-ups, and shareholder owned corporations.

**Figure 13.**
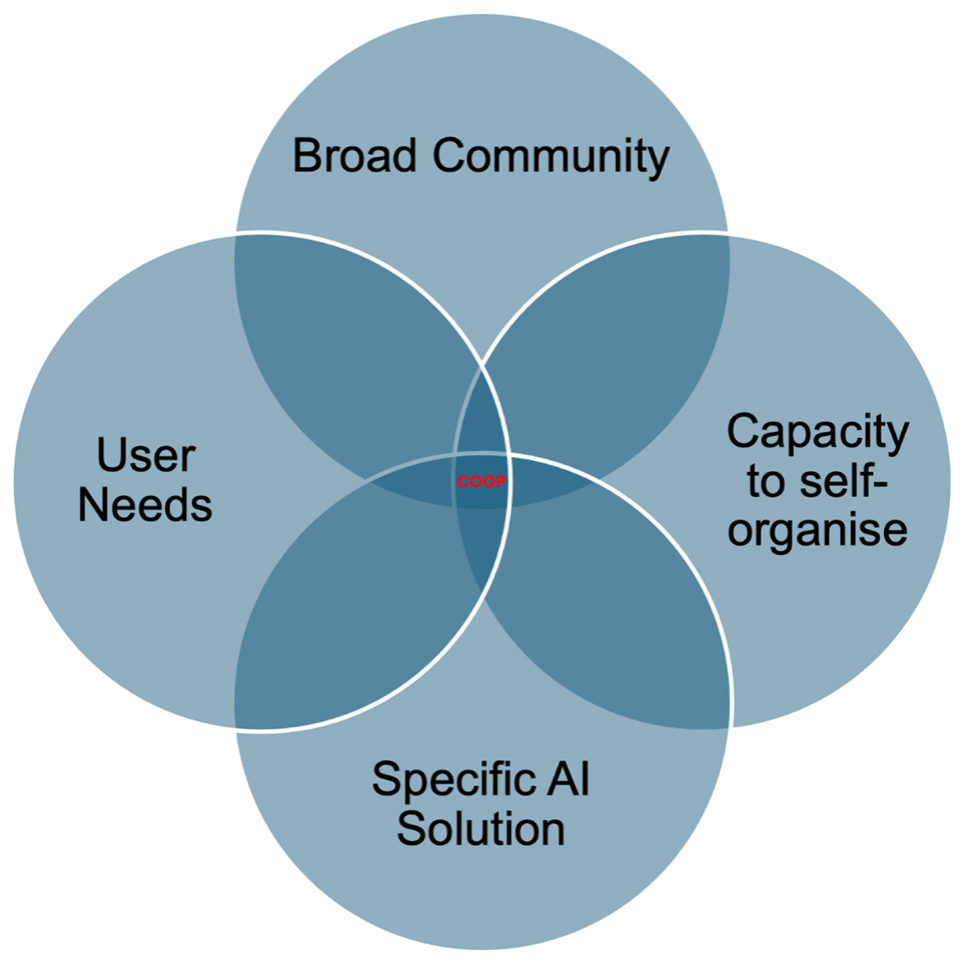
Intersectional aspects underpinning a potential AI cooperative.

There are practical recommendations that can support the development of cooperative AI infrastructures. In higher education environments, including Business Studies, but also in Computing Science, Digital Humanities, Library and Information Science, and Digital Social Science, we need to ensure that we incorporate learning and teaching about cooperative technologies, and their potential for prioritising the sustainability of digital infrastructure. Individuals interested in understanding more about this area should seek out their local platform cooperatives, and actively support them, to learn more about the cooperative model and their governance mechanisms. We encourage those running or co-owning platform cooperatives to publish their history, trajectories, obstacles, and achievements to provide further evidence and roadmaps which may support others. This may be done in collaboration with academic communities, and we would welcome further published studies of the co-owners and users of platform cooperatives, and how this model benefits them. We also recommend that those wishing to establish their own digital, platform, or AI cooperative should thoroughly explore a variety of aspects in advance, to ensure that this business model is appropriate (see Figure 13).

We acknowledge that READ_COOP is a highly unusual platform cooperative: in the provision of its AI tools, in its response to a specific sector; in the fact that it emerged from an incredibly well-resourced pair of European Union research grants, supported initially by a variety of university infrastructures and communities; and benefiting from both first-mover advantage and combined network affect which may create significant barriers to entry for competing platforms, potentially leading to a quasi-monopolistic position. This may make its success highly unusual: however, we believe there are several factors that can be taken into account when considering whether a platform cooperative would be a successful legal framework in which to embed AI.

Firstly, the scope and size of the platform’s specific potential community should be defined, as well as the community’s ability, capacity and desire to contribute to a shared infrastructure: the context it operates in is of utmost importance, and that should be well defined and understood. Secondly, ascertaining if the specific AI tool meets an ongoing, shared community need, responsive to general and common requirements, which will make a tricky technological task easier to accomplish, is crucial to the success of any platform, digital, or AI cooperative endeavour: the platform needs to respond to unmet needs, but with a task generic enough to demand broad community support. Thirdly, the availability of resources required for development, deployment, ownership, and operationalisation of the specific AI tool to meet these community needs will also be a crucial consideration, and the resources needed to undertake this development should not be underestimated. Fourthly, understanding the capacity and will for this community to self-organise into a cooperative society, including the source of the labour required (some of which will need to be voluntary, particularly in the start-up phase), will lead to realistic conversations about the establishment of a future AI cooperative. Finally, in the case of READ-COOP, the cooperative structure has supported community engagement, and the readiness of communities to be involved with platform development should be ascertained. The success of READ-COOP indicates that although the area of overlap where an AI cooperative can thrive is small, it can be fruitful: although fulfilling these criteria is difficult, and the success of the READ-COOP may have to stand as an outlier in this cooperative space.

We also acknowledge limitations of this study: our case study may not represent the wider technological world, given it is a bounded community; we concentrated on surveying Members, and have yet to survey Users; the Members who did respond to our queries were a subset self-selecting early adopters; and that the community-building and engagement we describe is also an effective marketing and market-growth strategy. Undertaking a structured study that compares our approach and business model to the community engagement undertaken by other commercial and non-commercial HTR platforms will also offer useful insights.

## 8. Conclusion

This paper has reported on the establishment and activities of READ-COOP, the cooperative structure that maintains and further develops Transkribus, the award-winning AI based Automated Text Recognition platform for historical documents. Since its founding in 2019, the cooperative has built a network of 227 highly engaged Members from over 30 countries internationally, that have financially, intellectually, and practically contributed to a machine-learning structure that has processed 90m digital images of historical documents, supporting 235,000 active Users. Using both Reflection-In-Action and qualitative questionnaire approaches, we have documented the history of READ-COOP between 2019 and 2024 and undertaken a community survey. This methodological framework elucidates novel insights into the benefits that an AI cooperative can provide for both Members and innovation in the infrastructure itself, grounding this analysis with Member feedback. Despite some concerns about costs, and approaches to open research, most Members of READ-COOP acknowledge considerable advantages, such as Member discounts, networking opportunities, technical assistance, and the ability to shape Transkribus’ development. The involvement of Members also benefits READ-COOP, including understanding the needs of the community, gathering input on technical development plans, designing and beta-testing new features, and democratic decision making regarding cooperative management. In describing this, we demonstrate that a cooperative can support the research and development in new technology, if it is established particularly with and for this context. READ-COOP Members are far more engaged in cooperative governance than is usual in other coops, indicating their suitability for technology innovation support ( Davis & Donaldson, 2000, 160).

It is our hope that in describing the Transkribus and READ-COOP journey, from our establishment as university-based EU funded research projects, to our independent commercial ventures between 2019 and 2024, that we have been able to communicate both the benefit of a cooperative technology infrastructure to a company and its members, but also the suitability of cooperative principles to the sustainability and governance of AI-based technology, although we acknowledge the unique set of circumstances underpinning Transkribus’ development. Using READ-COOP as a case study provides an opportunity to discuss wider issues regarding the business models which underpin AI, demonstrating that cooperative principles could provide an alternative mechanism for building trustworthy, responsible, and sustainable AI, in responding to specific sectoral needs. This impact extends beyond traditional metrics of success, in structurally embedding community input and ownership into the development process, fostering a sense of investment and responsibility among its members, and an accountability of the governance structure, while also providing an alternative set of economic values, that are often absent in conventional and dominant modern business models. We suggest that a cooperative route provides a sustainable path forward for other AI and ML initiatives, especially those developed through public funding. We suggest that any consideration of responsible and trustworthy AI must examine the underlying economic models and business practices of any given platform, and its ability to function cooperatively rather than competitively, to understand how its primary motivation and foundational purpose aligns with (or cannot align with) ethical principles. We must dream differently to operationalise responsible and transparent AI, otherwise it is already “too late” (RCM18).

A change towards cooperative governance for AI and ML could represent a significant shift in how we conceive of, innovate, and implement AI systems globally, advocating for a more democratized and equitable technology landscape. Platform cooperatives have the potential to build truly inclusive innovation communities: building relationships, which enact inclusive decision-making, with shared investment in technology, and the sharing of success and prosperity. It is these cooperative aspects that lead to technological democratisation. However, such an opportunity requires recognition and support from funders, universities, technology business incubators, policymakers, and the research community itself, in a reframing of economic success towards community-based governance, shared prosperity, and sustainability. At the moment, Transkribus is the only AI platform that we know of which has been established under cooperative principles, and it may be unique in its endeavours because of the privileges it enjoyed in its research funded, university supported research and development phase, its first-to-market advantage, the specific, networked community it supports, and in its technological research and develop endeavours. However, we hope that documenting and considering READ-COOP activities will allow others to consider a cooperative approach for establishing, innovating, sustaining, and growing trustworthy and reliable AI based tools, products, and services.

## Ethics statement

This research has had research ethics approval via the READ-COOP board (October 2023) and University of Edinburgh (School of Languages, Literatures and Cultures) Research Ethics Board processes (October 2020). Informed consent was given by survey participants for publication of anonymised results, this was obtained via the initial question (check box) of the online survey collection instrument.

## Data Availability

There is no data which underpins this research paper that can be made publicly available given GDPR constraints: all available datasets regarding READ-COOP are available via https://readcoop.eu. The paper is an overview of the business activities undertaken by the READ-COOP SCE. Data about Members and Users is therefore commercially and ethically sensitive from a GDPR perspective. No data processing or algorithmic approaches are discussed in the paper and therefore data is not associated to the research methods.

## Footnotes

^1^ Although many transcription platforms set out to deliver Handwritten Text Recognition (HTR), the term ATR (Automated Text Recognition) is now used to describe their activities given that print materials are also covered, see Hodel (2022) for a discussion on this shift in language.

^2^ These include: Aletheia ( https://www.primaresearch.org/tools/Aletheia) ( Clausner *et al.*, 2014); eScriptorium ( https://escriptorium.rich.ru.nl) ( Kiessling *et al.*, 2019); Kraken ( https://github.com/mittagessen/kraken) ( Kiessling, 2025); Leo ( https://www.tryleo.ai), Loghi ( https://github.com/knaw-huc/loghi) ( van Koert *et al.*, 2024); Monk ( https://monk.hpc.rug.nl/monk/demo.html) ( Schomaker, 2016); OCR4all ( https://fortext.net/tools/tools/ocr4all) ( Reul *et al.*, 2019); Teklia ( https://teklia.com) including its PyLaia ATR library ( https://gitlab.teklia.com/atr/pylaia) and Arkindex platform ( https://doc.teklia.com/arkindex/) ( Tarride *et al.*, 2024), and Callico tool ( https://teklia.com/our-solutions/callico/) ( Kermorvant *et al.*, 2024); TranSKriptorium ( http://transkriptorium.com which also emerged from partners in the READ project consortium) ( Sánchez *et al.*, 2024); and Verleden Tekst ( https://www.verledentekst.nl).

^3^ TranScriptorium, Project ID 600707, FP7-ICT-2011-9, European Commission (2015). See https://web.archive.org/web/20121201003048/http://transcriptorium.eu/, captured 1 ^st^ December 2012.

^4^ See https://web.archive.org/web/20150609184131/https://transkribus.eu/Transkribus/, captured June 9 ^th^ 2015.

^5^ Recognition and Enrichment of Archival Documents (READ), Project ID 674943, H2020-EINFRA-2015-1 European Commission (2016), https://read.transkribus.eu/

^6^ HTR and ATR systems use Character Error Rate to accurately measure output quality. Measuring and openly stating system performance in this way is more effective than sharing technical specification of models (such as parameters, or data storage size, which are often the only way that generative AI companies describe their systems, while withholding benchmarking metrics: there is a “significant lack of standardization in responsible AI reporting” ( AI Index, 2024, 5)).

^7^ See https://web.archive.org/web/20190410062144/https://read.transkribus.eu/network/ captured March 6 ^th^ 2019.

^8^ See Macmillan and Cusworth (2019); McKillop *et al.*, (2020); Ferreri and Vidal (2022); Noble and Ross (2021).

^9^ Examples include MIDATA, a not-for-profit established in 2015 by Swiss researchers, supporting users control of their own health data ( https://www.midata.coop/); and PescaData, a social enterprise improving data collection, communications and financial decision-making among small-scale fishing organisations in Latin America and the Caribbean ( https://pescadata.org).

^10^ For example, Brightest’s sustainability software platform uses AI-powered recommendation and productivity tools, see https://www.brightest.io. The LBMX cooperative offers real time analytics for buying groups and purchasing cooperatives, see https://www.lbmx.com/. However, it is not clear who provides their AI tools.

^11^ Throughout 2023, although there was a decline in AI private investment overall, “funding for generative AI surged, nearly octupling from 2022 to reach $25.2 billion” ( AI Index Report, 2024, 5).

^12^ At time of writing (October 2024) there are a few non-profit AI companies in operation. AllenAI ( https://allenai.org/) is “a non-profit research institute founded in 2014 with the mission of conducting high-impact AI research and engineering in service of the common good” ( AI2, n.d.) EleutherAI ( https://www.eleuther.ai/) “is a non-profit AI research lab that focuses on interpretability and alignment of large models” ( EleutherAI, 2023). Both are dependent on external resources, whether that is philanthropy (in the case of AllenAI) or access to processing infrastructure, which is how the community research group EleutherAI began, before formalising as a nonprofit foundation in 2023 funded by donations and grants ( Wiggers, 2023). There is also much activity happening in the open-source AI movement, such as LLM360 ( https://www.llm360.ai) which provides “fully accessible, open-source LLMs… Together, we can make AI research more open, transparent, and trustworthy” ( LLM360 Team, 2023), and Nomic ( https://www.nomic.ai), who state that “it’s critical to maintain a vibrant, collaborative, open-source ecosystem… freely accessible code, datasets, documentation, and model weights are all crucial for AI auditability and explainability”. Nomic raised investment to support their activities ( Nomic, 2023), and the commercial business model for open-source software development is well understood, sitting together with more ethical and responsible approaches to software and platform development ( Shahrivar *et al.*, 2018). Together ( https://www.together.xyz) is another for profit operating in the open-source AI space, again using cooperative language: “We build everything with the purpose of benefiting society” ( Together, 2023). However, for the most part, different, non-pure-profit approaches to AI infrastructures remain rare.

^13^ The six declarations including in Floridi and Cowls’ analysis are the Asilomar AI Principles, developed under the auspices of the Future of Life Institute (2017); The Montreal Declaration for Responsible AI developed under the auspices of the University of Montreal (2017); The General Principles offered in the second version of *Ethically Aligned Design: A Vision for Prioritizing Human Well-being with Autonomous and Intelligent Systems* ( IEEE, 2017); The Ethical Principles offered in the *Statement on Artificial Intelligence, Robotics and ‘Autonomous’ Systems,* published by the European Commission’s European Group on Ethics in Science and New Technologies ( EGE, 2018); The ‘five overarching principles for an AI code’ offered in UK House of Lords Artificial Intelligence Committee’s report, *AI in the UK: ready, willing and able?* ( House of Lords, 2018); and The Tenets of the Partnership on AI ( Partnership on AI, 2018).

^14^ Some of the declarations have vague nods to cooperative principles. The Asilomar Principles include the need for both “shared benefit” and “shared prosperity” from AI ( Future of Life Institute, 2017). IEEE acknowledges that “Measuring and honoring the potential of holistic economic prosperity should become more important than pursuing one-dimensional goals like productivity increase or GDP growth” ( IEEE, 2017, p.2). EGE states that “A spirit of global cooperation and public dialogue” ( EGE, 2018, 17) is required for regulation of AI development and application. Tenets explains that it will “remove ambiguity by building a culture of cooperation” ( Partnership on AI, 2018). However none of these declarations suggest any vehicles for doing so, nor do they interrogate underlying business or community support models.

^15^ There is a need for further work to undertake a similar mapping of a range of business models and activities to responsible AI principles, such as B-Corps (for-profit corporations certified for their social impact), and 501(c) non-profit organisations, unpicking the very nature of businesses and how their economic mechanisms affect their approach to responsible and transparent AI.

^16^ FP7-ICT - Specific Programme "Cooperation": Information and communication technologies ( https://cordis.europa.eu/programme/id/FP7-ICT/en). ICT-2011.8.2 - ICT for access to cultural resources ( https://cordis.europa.eu/programme/id/FP7_ICT-2011.8.2/en).

^17^ H2020-EU.1.4. - EXCELLENT SCIENCE - Research Infrastructures ( https://cordis.europa.eu/programme/id/H2020-EU.1.4./en). H2020-EU.1.4.1.3. - Development, deployment and operation of ICT-based e-infrastructures ( https://cordis.europa.eu/programme/id/H2020-EU.1.4.1.3./en). EINFRA-9-2015 - e-Infrastructures for virtual research environments (VRE) ( https://cordis.europa.eu/programme/id/H2020_EINFRA-9-2015/en).

^18^ The document upload is NL-AhGldA_2000_4787, Landdags- en kwartiersrecessen met bijbehoorende stukken. 1696, from the Gelders Archief Arnhem ( https://permalink.geldersarchief.nl/80A31735FD2848628582F7558E8DCA67). No known copyright.

^19^ Friedrich Wilhelm Raiffeisen (1818–1888 was a German banker and pioneer in the cooperative movement, playing a key role in the establishment of rural credit unions. Various cooperative banking structures now bear his name ( Deutscher Raiffeisenverband e.V., n.d.).

^20^ https://www.h2020.md/en/content/europe-changing-world-inclusive-innovative-and-reflective-societies

^21^ See https://readcoop.eu/wp-content/uploads/2020/05/Statutes_READ_COOP_SCE_current.pdf for current versions of the statutes.

^22^ https://single-market-economy.ec.europa.eu/sectors/proximity-and-social-economy/social-economy-eu/cooperatives/european-cooperative-society-sce_en

^23^ See Tsai *et al.* (2014) for an overview of SaaS benefits and challenges.

^24^ In May 2025, Günter Mühlberger concluded his term as Chair, and Andy Stauder concluded his term as Managing Director. Florian Stauder and Michaela Prien were appointed as Co-Executive Directors and C. Annemieke Romein became Chair of the Board: all are Members of READ-COOP.

^25^ The document upload is NL-AhGldA_2000_4787, Landdags- en kwartiersrecessen met bijbehoorende stukken. 1696, from the Gelders Archief Arnhem ( https://permalink.geldersarchief.nl/80A31735FD2848628582F7558E8DCA67. No known copyright.

^26^ Originally at https://readcoop.eu/readsearch/, all content is now at https://www.transkribus.org/sites.

^27^ One of the key improvements to TrOCR was the optimization of image ingest, allowing for more efficient and accurate processing. Additionally, the training data was expanded by incorporating roughly 180,000 pages of handwritten material into the ground truth set, significantly enhancing the model’s ability to recognize diverse handwriting styles, resulting in improved results in text recognition within Transkribus.

^28^ Ground Truth refers to a form of data that adheres to specified standards and is considered, at least by a group of people, to be an accurate representation of the material, in this case, handwritten or printed material ( Muehlberger *et al.*, 2019, 957)).

^29^ http://transkriptorium.com

^30^ Compare READ-COOP activities with the lack of true involvement with the likes of Google: their idea of operationalising AI accountability is merely to elicit feedback via an online form that has only one question, which is buried in their website footer https://ai.google/responsibility/principles/.
