# Supplementary material for: The artificial intelligence cooperative: READ-COOP, Transkribus, and the benefits of shared community infrastructure for automated text recognition
Source: Open Res Eur. 2025 Oct 7;5:16. Originally published 2025 Jan 20. [Version 2] doi: 10.12688/openreseurope.18747.2 (PMC12639283; doi:10.12688/openreseurope.18747.2)
Supplement: Peer review history

# Reviewer report: Reviewer response for version 2

**DOI:** 10.21956/openreseurope.22874.r61769

**Published:** 2025-11-21

**Authors:** Thibault Clerice

**Affiliations:** 1 Inria, Paris, France

Thank you for the careful and thorough revisions. We are satisfied that the majority of our concerns have now been addressed, and the manuscript is significantly strengthened as a result.

 We would, however, still have appreciated a finer-grained description of the status of the respondents to the survey---particularly with respect to their roles, engagement levels, or the length of their Membership---since these factors may have shaped their perspectives and thus influenced the results. Even a brief acknowledgement of such potential response biases would further contextualise the findings.

 Regarding our earlier comment on the references to the Horizon Impact Award, our concern was primarily raised in contrast to the former lack of proper citation of competitor initiatives, which created an imbalance in the original manuscript and could have been perceived as promotional. We are pleased to see that the citation strategy has now been adjusted, and this has resolved the issue.

 On the topic of Transkribus Sites, we understand and acknowledge your explanation of its purpose and added value for users. Still, we remain of the view that, regardless of intent, the feature does function as a form of retention mechanism unless users are able to export a static or dynamic code base enabling completely independent hosting beyond the platform. This may be worth further clarifying in future discussions of user autonomy.

 Finally, concerning the parallel with OpenAI: our initial observation centred specifically on questions of data provenance and the use of private data for building broader models. This remains a crucial consideration in contemporary AI governance, and even more so within the humanities, where visibility of research outputs and transparency of data usage are paramount. The point was especially salient given that concerns on this topic have previously been raised in the literature by Transkribus collaborators. That said, the revised phrasing now presents the business model and governance structures more transparently, which we appreciate and regard as a meaningful improvement.

 In summary, the manuscript is markedly clearer, more balanced, and more substantiated than in its previous version. We thank the authors for their diligence in responding to the feedback and believe the paper is now considerably strengthened.

If any, are all the source data and materials underlying the results available?

No

Is the work original in terms of material and argument?

Partly

Is the work clearly and cogently presented?

Yes

Is the argument persuasive and supported by evidence?

Partly

Does the research article contribute to the cultural, historical, social understanding of the field?

Yes

Does it sufficiently engage with relevant methodologies and secondary literature on the topic?

No

Reviewer Expertise:

Automatic Text Recognition, Open Data, Fair Data, Open Source, Digital Humanities, Computational Humanities

I confirm that I have read this submission and believe that I have an appropriate level of expertise to confirm that it is of an acceptable scientific standard.

# Reviewer report: Reviewer response for version 2

**DOI:** 10.21956/openreseurope.22874.r61768

**Published:** 2025-10-25

**Authors:** R. Trebor Scholz

**Affiliations:** 1 The New School, New York, New York, USA

I am satisfied with these changes and think that the paper can move ahead.

If any, are all the source data and materials underlying the results available?

Yes

Is the work original in terms of material and argument?

Yes

Is the work clearly and cogently presented?

Yes

Is the argument persuasive and supported by evidence?

Partly

Does the research article contribute to the cultural, historical, social understanding of the field?

Yes

Does it sufficiently engage with relevant methodologies and secondary literature on the topic?

Partly

Reviewer Expertise:

NA

I confirm that I have read this submission and believe that I have an appropriate level of expertise to confirm that it is of an acceptable scientific standard.

# Reviewer report: Reviewer response for version 1

**DOI:** 10.21956/openreseurope.20281.r50056

**Published:** 2025-02-19

**Authors:** Thibault Clerice

**Affiliations:** 1 Inria, Paris, France

**Introduction**

 The paper presents, explains and analyzes the choice and its aftermath behind the structural change from a publicly funded project (Transkribus) to a cooperative-based ownership of the infrastructure and developments. It uses the authors' own experience and their own company as a use case to defend the hypothesis that cooperative structuration in niche AI applications—but even more for research projects after public funding phases is a way to ensure its survival and sustainability. The authors present the cooperative status of READ-COOP as the element responsible for the success of the platform (Page 2, "This success [number of documents and users] is due to the cooperative approach...").

 After carefully reviewing the paper, I find that it effectively presents the argument that the cooperative structure has, for the time being (as the authors rightly acknowledge on page 29), ensured the short- to mid-term sustainability of the tool originally funded by public funds. However, the paper lacks sufficient argumentation and supporting sources to substantiate the claim that the company structure is responsible for the platform's success (measured in terms of the number of users, documents, or citations, as referenced in section 5.6 by the authors themselves). Furthermore, the paper appears to omit critical perspectives on this structural change and fails to appropriately cite the work of competitors. At times, the paper reads more as an advert for the cooperative (for example, the fact that Transkribus won an award is mentioned three times and cited twice in the bibliography) rather than a comprehensive analysis of this structural shift. In the following report, I will suggest additional avenues that I believe the authors should consider integrating into their paper in order to better represent the impact of the cooperative structure, its benefits, and its consequences on the community.

 Finally, the very small participation rate in the survey, along with its imbalance toward “founding” members (35% from 2019 and 56% from 2019 and 2020), warrants a more cautious interpretation of the results. For instance, when analyzing responses indicating that the primary motivation behind shareholding was the discount, it is important to consider how many of these respondents are recent members. Conducting an additional survey focused specifically on users would provide valuable insights and further strengthen the overall claims and conclusions of the paper. If institutions and individuals become members for discount rather than for cooperation, the same way they pay for subscriptions, how does the argument of the paper hold?

 I would like to conclude the introduction to my review by expressing my appreciation for the transparency demonstrated in this paper and its intent to provide a detailed perspective on the transition process that Transkribus underwent. It is undoubtedly challenging to write about the success of one’s own project, especially when it has been nurtured for over a decade. However, this close connection to the project can—as is the case here—limit the necessary critical perspective on practices that are genuinely aimed at supporting the community but also function as significant marketing and user retention strategies.

 This paper should be revised and resubmitted to address the issues I think are too big for a "Approve with reservations".

**I Academic good practices**

 Competing platforms are not appropriately cited, and works that should be referenced through formal citation are too often merely linked via URLs. All competitors are mentioned only by hyperlink—including one that is clearly incorrect ( www.scriptorium.com, which leads to a company specializing in content solutions and has no connection to ATR). Given the nature of some of the projects, specifically eScriptorium which is designed as a decentralized tool, citing their publication would be more meaningful than choosing a random instance. Similarly, HTR-United is referenced only by URL rather than through proper citation. Given that the authors also measure their own success through citation counts, it would be fair and academically rigorous to cite the relevant papers describing these competing platforms. Additionally, while Teklia is mentioned, the authors should also cite Callico, the tool that they produced.

 From the perspective of the paper, a more thorough analysis of (potentially non commercial) competitors within the same niche would strengthen the argument regarding the cooperative model, providing a clearer context for the discussion.

**II Terminology issues and ambiguities**

 I would have liked the authors to have defined and used the terms “users”, “members”, and “community” more precisely, as they appear to have distinct meanings: a member holds a share in the cooperative, users have accounts on the platform, and the community seems to encompass all users, whether institutional or individual. For example, on page 28 of the PDF, the term "READ-Coop users" is used, but in this context, it likely refers to members. The term “shareholder” may be more appropriate in such cases, as the use of “member”—which can refer either to a member of the cooperative or to a member of the broader community—introduces ambiguity.

 A similar issue in terminology occurs on page 22 of the PDF. The paragraph begins by mentioning openly licensed ground truth (i.e., data) but then abruptly shifts to discussing openly licensed models. This sudden transition is confusing, particularly since open data is not addressed until the middle of an already lengthy paragraph. Moreover, the term “open model” is debatable in this context, as these models are only accessible within the platform's infrastructure and, in some cases, restricted to specific subscription tiers (e.g., Faucon Français and potentially other similarly sized models are limited to Scholar users). In AI discourse, open models typically refer to models with publicly available weights. A more precise term would be shared models or internally available models. Here, I would argue that these models are equally or less “open” than OpenAI ones.

 In the same paragraph of 5.6 Impact, the phrase “102 models available via HTR-United in October 2024” should instead refer to datasets, as HTR-United only provides training data rather than pre-trained models.

 Similarly, an issue with terminology appears on page 18, where a 5% CER (Character Error Rate) is mentioned. It should be clarified that, to our knowledge, Transkribus reports only development-set scores, not test-set scores. While this provides a useful metric, it may also indicate overfitting to a specific development set. Explicitly stating this distinction would improve the paper’s precision and the fairness of comparison with other tools.

 On page 20, the paper mentions AI-based search functionalities. However, these are not clearly defined (e.g., are they referring to vector search or document embeddings?). The term “AI-based search functionalities” is somewhat vague and could be perceived as marketing language, so it would be beneficial to specify the underlying techniques for greater clarity and academic rigor.

**III Success of Transkribus and relationship to the cooperative structure**

 I recommend the paper to acknowledge or address three mechanisms it benefited from or put in place to ensure its position as the main platform.

III.a Addressing the First-Mover Advantages (FMA) and/or First-Mover Benefits regarding the success of the platform

 The paper does not draw all the consequences of the fact that Transkribus was “the first handwritten text recognition (HTR) platform to provide access to the power of ATR without requiring users to set up or manage their own machine learning or AI infrastructure.” In the context of an economic analysis of the platform’s structuration and the broader claim that this structuration is responsible for its success and its number of users or members, the paper should at least acknowledge and discuss the role of First-Mover Advantages (FMA) or First-Mover Benefits (FMB) (Kerin et al. 1992 [Ref-2]; Cirik & Makadok 2023 [Ref-1]). (Cha 2013 [Ref-4]; Papachristos et al. 2022 [Ref-3]) have demonstrated that such advantages—when combined with effective user retention mechanisms—tend to foster monopolistic tendencies. Platforms such as MySpace, Facebook, Twitter, Instagram, and TikTok have all benefited from these dynamics.

*Potential bibliography:*

 - Kubilay Cirik and Richard Makadok, 2023 [Ref-1] : First-Mover Advantages versus First-Mover Benefits: What’s the Difference and Why Does It Matter?. AMR, 48, 409–431, https://doi.org/10.5465/amr.2017.0499

 - Kerin, R. A., Varadarajan, P. R., & Peterson, R. A. (1992) [Ref-2]. First-Mover Advantage: A Synthesis, Conceptual Framework, and Research Propositions. Journal of Marketing, 56(4), 33-52. https://doi.org/10.1177/002224299205600404

 - Papachristos, G., & Suarez, F. (2022) [Ref-3]. First Mover, Fast Second or Later Mover in Platform Industries? An Integrated Model of Entry Timing Advantages. *An Integrated Model of Entry Timing Advantages (June 15, 2022)*.

 - Cha, J. (2013) [Ref-4]. Examining First Mover Advantages among Social Networking Sites. *Media Management and Economics Research in a Transmedia Environment*, *1*(1), 2-9.

III.b Addressing Network Effects

 Network effects—where the value or utility of a service increases with the number of users—clearly contributed to Transkribus’ success and should not be overlooked in the analysis of its growth. This phenomenon is particularly significant in a niche market such as Automated Text Recognition (ATR) for historical documents, where the user base, primarily composed of GLAM institutions and scholars, is tightly connected and partially interdependent.

 The adoption of Transkribus can easily propagate through academic networks. For instance, a research grant involving a team that uses Transkribus may indirectly encourage or directly force its adoption by partner institutions, especially given that trained models are not exportable outside the platform. The inherently collaborative nature of grant-funded research in contemporary academia amplifies these network effects, reinforcing user retention. Moreover, in combination with FMA and FMB, these dynamics may create significant barriers to entry for competing platforms, potentially leading to a quasi-monopolistic position from which users find it difficult to switch.

 The network effect is clearly observable in the membership base in Germany, Austria, and the Netherlands, as noted by the authors in reference to the original network that helped build Transkribus.

III.c Addressing free use as a marketing tools

 In multiple instances, the authors present free credits as a means to “allow trial and support hobby users,” to “assist training events and students in their studies,” and to “promote diverse research.” We do not dispute the accuracy of these statements. However, in the context of a paper that leans toward management studies, it seems inadequate not to also analyze these free credits as marketing tools (Li 2022 [Ref-5]). When combined with network effects and the closed nature of both the software and models, this strategy functions as a potentially effective user retention mechanism.

 This is one example where the authors’ proximity to the subject—their own company and project—may lead to an incomplete analysis of the business mechanisms necessary for a company’s sustainability. A more critical discussion of these strategic choices would contribute to a more comprehensive and balanced assessment.  

*Potential bibliography:*

 -  Li, H. (A.) (2022 [Ref-5]). Converting free users to paid subscribers in the SaaS context: The impact of marketing touchpoints, message content, and usage. Production and Operations Management, 31, 2185–2203. https://doi.org/10.1111/poms.13672

**IV. READ-Coop, Transparency and cooperative as an alternative that pushes in the direction of more transparent and more responsible AI.**

 READ-Coop positions itself as an alternative to the practices of large AI companies. The argument that READ-Coop reinvests its profits into infrastructure rather than prioritizing shareholder returns is well-founded and clearly articulated. However, several points raised by the authors appear to be overly favorable to their own company:

1. While Transkribus does not sell private user data directly to third parties, it does sell enhanced models trained on aggregated user data, including to commercial entities. Although the profits from these sales are reinvested in the company, this practice bears similarities to what OpenAI does—leveraging user interactions to improve its models, which are then commercialized. In both cases, neither the models nor the underlying data are made freely available. Given this parallel, how does READ-Coop's approach fundamentally differ from that of OpenAI?
2. Unlike companies such as META, and even more so like OpenAI, READ-Coop does not release the weights of its models or the software necessary for their use. Given that the paper advocates for greater transparency in AI, it does not sufficiently address this resemblance between READ-Coop and the very actors from which it seeks to differentiate itself.
3. The paper mentions Teklia as an alternative to Transkribus; however, this framing is somewhat imprecise. Teklia, as a company, should rather be compared to READ-Coop, whereas Arkindex, its platform, serves as an alternative to Transkribus. While both are corporate entities, Teklia has embraced an open-source, open-weight, and open-tool approach—akin to companies such as Mistral and Pleias. A comparison with smaller AI companies, rather than solely with major technology firms (GAFAM), would likely strengthen the paper’s argument.
4. In relation to the previous two points regarding the openness of data and tools, another relevant issue is READ-Coop’s evolving terms of service. Until 2024, the platform’s terms and conditions explicitly allowed users to download their trained models (see https://webcf.waybackmachine.org/web/20241004060057/https://readcoop.org/terms , section 12.3.2: “You have the option to make your trained models available for general use by providing explicit consent. If you wish to make your Training Material publicly available along with the model via Transkribus, you must grant explicit consent for this specific purpose. You are free to export the model files of the models that you have trained.”). This clause has since been removed. To my knowledge, this option was never actually implemented, but given its significance, such a change in terms and conditions should have been explicitly communicated to users—yet no such notification appears to have been issued. In the context of the paper’s repeated emphasis on transparent AI, how do the authors analyze such modifications to its policies?

Finally, given these similarities, the paper does not sufficiently address concerns regarding the long-term security of Transkribus’ non-profit status. As the authors themselves note, OpenAI initially operated with a non-profit mission but later transitioned to a for-profit model. Given that Transkribus’ AI-related code is closed-source and its models are not downloadable, what mechanisms remain for users (as distinct from members) to trust that the underlying model and platform policies will not change in the future? Addressing this question would strengthen the discussion on transparency, governance, and long-term commitments to the community.

 Regarding transparent AI and best practices, as discussed in section 5.6 Impact, as well as the respectful acknowledgment of users' contributions, the current Transkribus platform does not provide any details about the datasets used to train its transformer models, such as Faucon Francais. This raises concerns both from an AI transparency perspective and from an academic standpoint, as it fails to properly recognize the contributions of data annotators (and the publication of one of the authors having focused on the need to rehabilitate the work of annotators, such as Father Busa's punch card operatives, should warrant for at least addressing the topic here). The gap between the stated commitment to transparency and its actual implementation warrants further discussion, particularly given the tension between READ-Coop’s dual role as an academic and transparent cooperative and its need to attract users and maintain a sustainable revenue stream. It also, to some extent, ignores the contribution of its own community from 2022 mentioned in 5.6 regarding the best practices to acknowledge the role of data in model training, which could show a more tenuous relationship between the company and its community.

**V. Survey’s limitations and additional data required**

 In the rest of the papers, some analyses are ignoring potential other reasons for the success of Transkribus, and the lack of data is leading to a lower capacity to admit the argument.

- “As of October 2024, 45% of READ-COOP members are private individuals (see Table 2), and the growth of the private membership option has been strongest over time, indicating how invested individual users have become in the operations of the cooperative, although overall voting share remains low.” Given the response to the survey, this analysis could be challenged by at least one competing hypothesis: could it be that these new member-users are significant users of the platform with limited institutional support, leading them to seek the private discount? Both hypotheses could be tested or refined through a better response rate to the original member survey, or by analyzing the percentage of users whose institutions are not members, thus potentially lacking access to institutional discounts. It would also be useful to identify users who self-identify as independent scholars, placing them in a position somewhat between institutional members and traditional members.
- The survey data have not been released. There is no explanation for why the survey data are not provided alongside the paper, especially since they are analyzed in depth. If the survey data cannot be shared due to privacy concerns, this should be explicitly mentioned to clarify why they are unavailable.
- The survey response rate is notably small. In the context of a paper advocating for the use of cooperative structures to foster “community ownership,” how do the authors account for the low number of survey responses, particularly if the community genuinely cares about the structure of READ-COOP?
- From an ethical standpoint, it is not clear whether the authors of the paper responded to the survey or if their institutions participated. As mentioned in the paper, at least one author is a founding shareholder of the cooperative. However, it is not specified whether the founding members or authors were permitted to answer the survey. While I would expect that this was not the case, this should be explicitly stated to ensure transparency.
- While it is known that Transkribus models are not downloadable, the paper does not address the sustainability of the new Transkribus Sites. It would enhance the paper to discuss whether users can easily export and host these sites themselves, or if this functionality is primarily designed as a user retention mechanism.

If any, are all the source data and materials underlying the results available?

No

Is the work original in terms of material and argument?

Partly

Is the work clearly and cogently presented?

Yes

Is the argument persuasive and supported by evidence?

Partly

Does the research article contribute to the cultural, historical, social understanding of the field?

Yes

Does it sufficiently engage with relevant methodologies and secondary literature on the topic?

No

Reviewer Expertise:

Automatic Text Recognition, Open Data, Fair Data, Open Source, Digital Humanities, Computational Humanities

I confirm that I have read this submission and believe that I have an appropriate level of expertise to state that I do not consider it to be of an acceptable scientific standard, for reasons outlined above.

## References

[1] : First-Mover Advantages versus First-Mover Benefits: What’s the Difference and Why Does It Matter?. *Academy of Management Review* .2023;48(3) : 10.5465/amr.2017.0499 409-431 10.5465/amr.2017.0499

[2] : First-Mover Advantage: A Synthesis, Conceptual Framework, and Research Propositions. *Journal of Marketing* .1992;56(4) : 10.1177/002224299205600404 33-52 10.1177/002224299205600404

[3] : First mover, Fast Second or Later Mover in Platform Industries? An Integrated Model of Entry Timing Advantages. *SSRN Electronic Journal* .2022; 10.2139/ssrn.4156876 10.2139/ssrn.4156876

[4] : Examining First Mover Advantages among Social Networking. *Media Management and Economics Research in a Transmedia Environment* .2013; Reference source

[5] : Converting free users to paid subscribers in the SaaS context: The impact of marketing touchpoints, message content, and usage. *Production and Operations Management* .2022;31(5) : 10.1111/poms.13672 2185-2203 10.1111/poms.13672

# Response

**Published:** 2025-08-27

**Authors:** Melissa Terras

**Affiliations:** Design Informatics, The University of Edinburgh Edinburgh College of Art, Edinburgh, Scotland, UK

Thank you for your close critical reading of the paper. You rightly note how hard it is to maintain a critical distance from something that you yourself have built, and your comments have helped us note where to take a step back and reframe our argument, and to provide further detail and evidence to strengthen it, which we have done in a revised version. We have added a section on how the cooperative operates internally, and hope that this demonstrates the mechanisms by which Members have privileged access to the development team, while contributing to transparent governance. We have clearly defined and differentiated Users and Members throughout, and we hope that means we have added clarity to the text so that the different roles are differentiated. We prefer to stick with the term Members – which we have now capitalised throughout– rather than shareholders as this is the terminology we use in READ-COOP business and statutes. To prove that we have exceptional engagement from our Members we have gone back to meeting minutes and official auditor records and have detailed in a new section a) the voting record of our Members in our official meetings and b) their attendance at monthly meetings. This is presented in a new section. We have stressed that while attendance at cooperative movement management meetings has been noted as being low in cooperatives across the world (Mohd-Saleh et al. 2025), with previous research showing “no matter what size of region 95% or more of cooperative members decline to be involved in their Society’s governance” (Davis and Donaldson 2000, 160), READ-COOP has exceptional levels of engagement with membership, with an average member attendance of 42%, and average voting rate of 55% (some members hold more than one vote). We have clarified throughout the text how the mechanisms that we have put in place for Members have contributed to technological development as well as community building. We hope that this clarifies and shores up our claims that our Members are indeed a core part of the READ-COOP, contributing to feature development and community, as well as promotion, and to access discounted use of the platform. I hope this allays criticism that Members only join READ-COOP for the discount: indeed our analysis of reasons for joining READ-COOP (Table 4) demonstrates that only 21% of our surveyed Members viewed this as a priority for joining READ-COOP, so the argument must go beyond accessing discounts. We also counter the fact that a small number of Members responded to the survey: survey response rates are generally 10% in the current survey fatigue climate and we have pointed out that our response rates provide confident estimates statistically, although we have also stressed that it is highly engaged Members who have responded.  We have made it clearer that our survey only canvassed Members, and that those who chose to respond were self-selecting early adopters and early Members. Unfortunately, it is not possible to also include a User survey in this paper given the wordcount limitations but we have made it clear that this will be returned to in a future study, which we always planned to do. We have also noted that a further analysis and comparison of our competitor platform’s liaisons with their members would also be worthy of an in-depth future study of its own: doing this properly would result in another paper and so we cannot include that analysis here. We believed that we were following the Journal guidelines on citation of sources, but we have taken on board that we could cite other’s publications as well as websites and so we have done that throughout. We apologies for the erroneous URL which must have crept in in the editing phase. We have added a discussion on first mover advantage, network effects, and SaaS marketing approaches to free initial access. We have set out our position on where we have, and have not, acknowledged data annotators to date, and the work we are doing to build better approaches to that, acknowledging this tension (which not only we are faced with). We also have to point out that we did mention the long term aims of the cooperative, and the fact we could not pivot to a more common business model (in the discussion section): we are not a not-for-profit, and have never been a not-for-profit, but a cooperative, and the co-ownership model means we cannot simply sell the business, nor raise funds from venture capitalists or other shareholder investors who wish to take a return on their investment – so we cannot do an “OpenAI” because of the way that we were legally established. It has been the point of the article to describe all of this, but we have added some more phrasing to that end given it did not land with the reader. There is criticism in the comments about Transkribus Sites, which again misreads and misrepresents our intentions. We have made it clear that all material in Transkribus—including transcripts, metadata, and document images—is fully exportable at any time in open formats (e.g. PAGE XML). Users are entirely free to build their own digital editions elsewhere, and many do. Transkribus Sites was created because many users, especially in academic and heritage contexts, struggle to host and present their material effectively—even after completing transcription work. For those users, Sites solves a problem that has gone unsolved for years: how to share work publicly without needing institutional infrastructure, technical support, or additional funding beyond yearly subscription costs: this is a convenience feature, not a retention strategy. Users who choose Sites do so because it makes their work visible, searchable, and citable with almost no overhead. The suggestion that this is primarily about user retention misreads both our intentions and the feedback we receive from the community. If anything, Sites has increased openness and visibility of materials that otherwise would remain stored in local folders, inaccessible to others. Sites is not a trap: it is an optional, appreciated tool for dissemination, and we have made this clearer in the text. We find the comment that our approaches are akin to what OpenAI is doing rather misleading: while we sell services based on aggregated models (Text Titan, etc), we don’t sell the model as such. We also openly communicate this to our owners, and anyone can join and become a co-owner, join internal meetings, and vote on how we build, use and commercialise the technology. We centralise as much as necessary to generate the necessary income to keep the cooperative infrastructure operational, and there is no way to earn a profit for any shareholder. We do have to maintain a level of closed-source models to sustain our business. In summary, we offer infrastructure with interoperable export functionality and hosted models, but we keep any data private that users do not      want to share, however, we openly communicate that private data may be used to generate models that benefit other users, and any stakeholder can join as a member with extensive rights. Data sharing is used to generate income, but we fundamentally believe we are operating in a very different way than OpenAI and it is rather disappointing that this hasn’t been understood, given the focus of our paper is to communicate the business model underlying Transkribus. We have tweaked this throughout in the hope that the reader will now understand what we are trying to communicate here, and that the Members have access to transparent governance, should they wish to engage.  Re Horizon Impact Award – we mention this in the abstract, and only once in the body of the text, and simply say we are “award-winning” in the conclusion. This is a normal level of mentions of any pertinent detail in any paper on any subject. We cite two different sources in the body of the text (a webpage and a video with different information in each, both produced by the EU) so we do not think that we are over promoting the fact that we won this major European prize. It is strange why a reviewer would criticise one properly cited mention in the body of the text of such an international award, or the fact that we mention we were “award-winning” in the conclusion, and so we must push back on the framing that mentioning the Horizon Impact Award, in a paper that is published in an EC funded journal, that details the exceptional impact an EC funded project has had, proves that the article is only an advert for us. On the contrary, we have included here much detail about the business operations of an AI cooperative which were not previously in the public domain, and we believe, at a time of growing concern about responsible AI governance, that documenting our founding, operations, and activities will give pause for thought about the business models that underpin all AI.

# Reviewer report: Reviewer response for version 1

**DOI:** 10.21956/openreseurope.20281.r50050

**Published:** 2025-02-18

**Authors:** R. Trebor Scholz

**Affiliations:** 1 The New School, New York, New York, USA

– Starting the paper with such a definitive conclusion may be overreaching. READ-COOP is an outlier in the cooperative landscape. Unlike most cooperatives, it applies AI/ML technologies as a tech cooperative for a niche product, which is then socialized through an institutional alliance or cooperative. While this is an effective way to engage stakeholders, it has also benefited from a substantial EURO 10.6 million grant while being associated with a university. These circumstances are not representative of the majority of cooperatives, even in the Global North. Relatedly, the claim that READ-COOP demonstrates that cooperatives can effectively maintain and govern AI platforms lacks sufficient context. A more specific, sector-focused claim would be more convincing. The paper suggests that AI platforms initially funded by grants can transition to financially self-sustaining cooperative structures, but greater contextual specificity would strengthen this argument. And this matters: The paper suggests that cooperative principles provide a sustainable governance model for AI platforms, as they are built on community involvement, ethical considerations, and public investment. READ COOP is a wonderful, successful example of a platform coop but it is also an aberration. It can't serve as a blueprint for the coop movement. The claim feels like an unhelpful overreach. It is successful but may not be the North Star for the coop movement as their needed products and co-op structures are vastly different.

 – The connection between cooperative or community-owned models and Schneider’s “Exit to Community” proposal is not entirely logical in terms of flow. Schneider’s concept does not align with what READ-COOP has done, making the comparison somewhat misplaced.

 – The reference to Pérotin (2021) linking cooperative governance to higher productivity is specific to worker cooperatives, not multi stakeholder cooperatives. Pérotin’s scholarship does not support this generalization, making the claim inaccurate.

 – Under Digital Infrastructure, the assertion that the SEWA Cooperative Federation (India) supports women's social enterprises with digital infrastructure is incorrect. SEWA has engaged in small-scale experiments with blockchain in a health services cooperative and has conducted several platform cooperative experiments in agriculture and delivery services. However, the delivery platform initiatives failed, and the agriculture-based experiment does not provide digital infrastructure in a meaningful way. Therefore, the claim that SEWA offers a robust digital infrastructure for women's social enterprises is factually inaccurate.

 – While cooperative approaches to digital infrastructure deserve further scrutiny, the most productive area of intervention may lie elsewhere. Historically, cooperatives have not been strong in R&D, not only due to funding constraints but also because of an aging membership base. Greater potential may exist with very large cooperatives, particularly cooperative banks, some of which generate billions in annual revenue. However, these entities often operate as cooperatives in name only rather than as genuinely justiceoriented organizations. Similar trends can be seen in the U.S. with examples like Cabot Cheese and large agricultural cooperatives, which function more like traditional corporations than democratic, member-driven enterprises.

 – Further clarification of READ-COOP’s structure would be useful. There are so many technical details but this is not clear. It is a cooperative of institutional members who use a product designed by a team within a university, which is part of the cooperative. It is unclear whether the developer team operates as an independent tech cooperative or if it functions under a different structure. Explicitly outlining the groups that make up the cooperative would provide greater clarity.

 – The democratic governance numbers seem too good to be true: 84% participated in monthly member meetings. On its own, this seems almost unbelievable. In larger co-ops, participation is often around 10%, even when considered successful. It would also be important to know how long members stayed in these meetings, as brief check-ins may not indicate deep engagement. If they stay for the meetings, this would be a huge success, almost unprecedented.

If any, are all the source data and materials underlying the results available?

Yes

Is the work original in terms of material and argument?

Yes

Is the work clearly and cogently presented?

Yes

Is the argument persuasive and supported by evidence?

Partly

Does the research article contribute to the cultural, historical, social understanding of the field?

Yes

Does it sufficiently engage with relevant methodologies and secondary literature on the topic?

Partly

Reviewer Expertise:

NA

I confirm that I have read this submission and believe that I have an appropriate level of expertise to confirm that it is of an acceptable scientific standard, however I have significant reservations, as outlined above.

# Response

**Published:** 2025-08-27

**Authors:** Melissa Terras

**Affiliations:** Design Informatics, The University of Edinburgh Edinburgh College of Art, Edinburgh, Scotland, UK

We thank you for this extensive and considered report, and have taken on board your critical suggestions fully in revisions, adding more nuance to our argument, toning down language that could be seen as over-reaching, and highlighting this study’s bounds and limitations, particularly with regards to the fact that READ-COOP SCE may be thought of as an cooperative outlier given its particular focus, context and circumstance. We have added a section on how the cooperative is structured. We have also used your comments to add clarity in places (for example stressing that Transkribus is no longer associated with a particular university, but it is an independent entity). We also thank you for the observation that most engagement with cooperative governance is low – having now added a section on Member engagement with the coop, after consulting our records, we can confirm that over the past 6 years we have an average member attendance of 42% to formal meetings, with an average voting rate of 55% (some members hold more than one vote). Attendance at informal monthly meetings was between 50 and 75 members attending (out of a growing list of between 209-255 members) throughout 2024. We have taken on the point that this may also demonstrate that the cooperative mechanism we established is supporting effective digital innovation and engagement with a core group of committed users. We have used this expert reading to improve the paper from its first submission and the next version now has far more nuance: thank you.
